# Integrative Single-Cell Transcriptomic Analysis of Human Fetal Thymocyte Development

**DOI:** 10.3389/fgene.2021.679616

**Published:** 2021-07-02

**Authors:** Yuchen Li, Weihong Zeng, Tong Li, Yanyan Guo, Guangyong Zheng, Xiaoying He, Lilian Bai, Guolian Ding, Li Jin, Xinmei Liu

**Affiliations:** ^1^International Peace Maternity and Child Health Hospital, School of Medicine, Shanghai Jiao Tong University, Shanghai, China; ^2^Shanghai Key Laboratory of Embryo Original Disease, Shanghai, China; ^3^Research Units of Embryo Original Diseases, Chinese Academy of Medical Sciences, Shanghai, China; ^4^Bio-Med Big Data Center, Shanghai Institutes for Biological Sciences, Chinese Academy of Sciences, Shanghai, China; ^5^Obstetrics and Gynecology Hospital, Institute of Reproduction and Development, Fudan University, Shanghai, China

**Keywords:** fetal thymus, human and murine, single-cell RNA-seq, T lymphopoiesis, transcriptional dynamics

## Abstract

Intrathymic differentiation of T lymphocytes begins as early as intrauterine stage, yet the T cell lineage decisions of human fetal thymocytes at different gestational ages are not currently understood. Here, we performed integrative single-cell analyses of thymocytes across gestational ages. We identified conserved candidates underlying the selection of T cell receptor (TCR) lineages in different human fetal stages. The trajectory of early thymocyte commitment during fetal growth was also characterized. Comparisons with mouse data revealed conserved and species-specific transcriptional dynamics of thymocyte proliferation, apoptosis and selection. Genome-wide association study (GWAS) data associated with multiple autoimmune disorders were analyzed to characterize susceptibility genes that are highly expressed at specific stages during fetal thymocyte development. In summary, our integrative map describes previously underappreciated aspects of human thymocyte development, and provides a comprehensive reference for understanding T cell lymphopoiesis in a self-tolerant and functional adaptive immune system.

## Introduction

T lymphocytes play indispensable roles in cellular immunity, defending against latent threats such as pathogens, exogenous grafts and neoplasms. Hematopoietic progenitors seeded in the thymus commit toward T cell lineage and undergo step-wise differentiation processes, including genomic recombination to form functionally mature T cell receptors (TCRs) and positive and negative selection to achieve central tolerance and maintain immune homeostasis. Based on their surface protein markers, thymocytes have been traditionally divided into three stages: the double negative (DN, CD4^–^CD8^–^), double positive (DP, CD4^+^CD8^+^) and mature single positive (SP, CD4^+^CD8^–^ or CD4^–^CD8^+^) stages (Spits, 2002). In response to chemokine signals, mature thymocytes emigrate to peripheral lymphoid tissues and organs through the blood circulation, which contributes to the heterogeneity of adaptive immunity (Halkias et al., 2014).

In humans, T lymphopoiesis usually begins in the embryonic and fetal phases when major hematopoietic progenitors are derived from the liver instead of the bone marrow, which dominates hematopoiesis from the second trimester to the postnatal stage (Jagannathan-Bogdan and Zon, 2013). The earliest mature T cells emerge in the thymus and periphery starting at weeks 10–12, which corresponds with the thymus organogenesis beginning at approximately 8 weeks of gestation (Farley et al., 2013). Distinct T cell lineages arising from fetal and adult hematopoietic progenitors have been reported, indicating that the molecular and functional features of T cell precursors shaped in the thymus can vary dramatically across developmental stages (Mold et al., 2010).

Based on the types of TCR chains, T cells are divided into αβ-T and γδ-T lineages which participate in distinct aspects of T cell immunity. For example, αβ-T cells mainly serve as the conventional helper or cytotoxic lymphocytes (Taniuchi, 2018), while γδ-T cells can have innate-like and adaptive immune functions (Lanier, 2013). However, both of these cell lineages need to undergo intrathymic differentiation, during which T cell precursors select either the αβ- or γδ-TCR lineages (Ciofani and Zúñiga-Pflücker, 2010). Although previous research has investigated the development of these two T cell lineages (Mingueneau et al., 2013; Sagar et al., 2020), the key factors that regulate the lineage decisions are still poorly understood.

Given the difficulty of obtaining human tissues *in vivo*, research on T cell development during gestation has usually been performed *via* animal experiments. However, the biological features of thymocytes are not always comparable between mice and humans. For example, the DN thymocytes in mice can be subdivided into DN1-DN4 thymocytes based on the surface antigens CD25 and CD44, which have not been used to classify early thymic progenitors (ETPs) in humans (Dik et al., 2005; Mingueneau et al., 2013). In humans, some thymocytes do not complete β-selection until DP stage, in contrast to the situation in mice whose immature single positive (ISP) thymocytes possess functionally rearranged TCRs (Taghon and Rothenberg, 2008). These limitations and differences demonstrate the requirement for comparative analyses of differentiating thymocytes in these two species.

Single-cell RNA sequencing (scRNA-seq) has recently been used to profile the cellular heterogeneity and dynamics of gene expression during thymic development in both humans and mice (Kernfeld et al., 2018; Zeng et al., 2019; Park et al., 2020). Bioinformatics methods allow biologists to integrate datasets across different batches, conditions, ages and even species to build large-scale atlases (Haghverdi et al., 2018; Stuart et al., 2019). However, data integration can sometimes incorrectly eliminate the biological variations due to developmental stages; thus its implementation in studies on fetal development remains controversial (Behjati et al., 2018; Hie et al., 2019; Tran et al., 2020). In this study, we performed scRNA-seq of developing human thymocytes at the fetal stage. Through integration analysis with publicly available data, we connected fetal growth with thymocyte differentiation. The common features related to TCR decisions were evaluated. Trajectory analysis characterized the lineage specification and protein maturation of fetal ETPs. Comparisons with mouse scRNA-seq data revealed the conserved and species-specific features of humans and mice. Finally, the potential links between human thymocyte development and autoimmune disorders were investigated at a single cell resolution.

## Results

### Developing Thymocytes Identified by scRNA-seq

**FIGURE 1:**
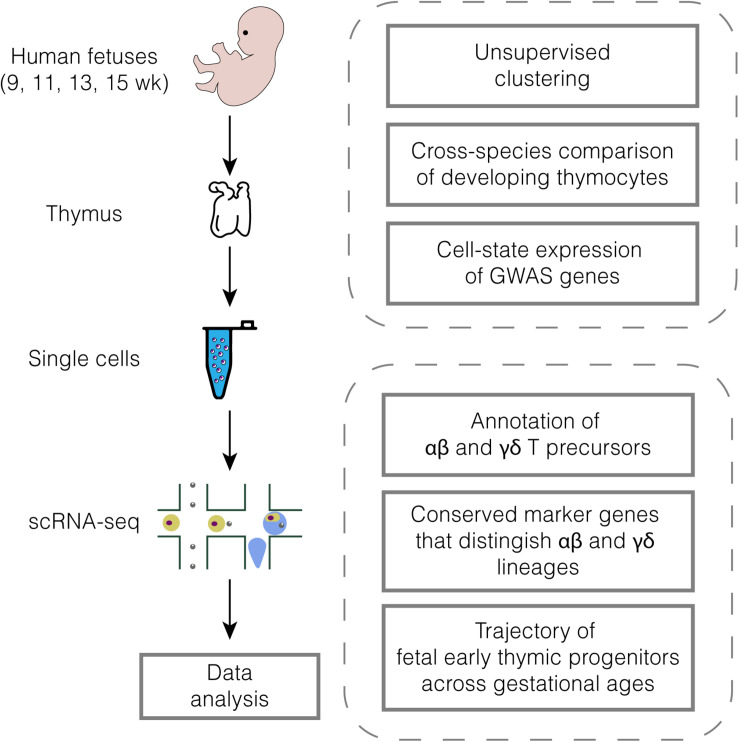
Diagram of the experimental workflow. All samples were processed immediately after elective medical termination.

To capture the transcriptomic signatures of developing human thymocytes, we isolated 7-aminoactinomycin D^–^ (7-AAD^–^) fetal thymus cells with fluorescence-activated cell sorting (FACS) from gestational weeks 9–15 and performed droplet-based scRNA-seq (10× Genomics Chromium System) (Zheng et al., 2017). The workflow of the analyses is briefly shown in Figure 1. After correction for contamination and removal of low-quality cells and doublets (Supplementary Figures 1A–E), we retained expression profiles for 21,155 cells (Supplementary Table 1). By initially clustering cells at a low resolution and annotating clusters with cell-type marker genes, we identified and discarded clusters with non-T cell lineages such as myeloid cells (*CD14*, *LYZ*, and *CLEC9A*) (Bassler et al., 2019), mast cells (*CPA3*, *TPSAB1*, and *TPSB2*) (Wernersson and Pejler, 2014), mesenchymal cells (*COL3A1*, *COL1A1*, and *PDGFRA*) (De Val and Black, 2009) and B cells (*CD19*, *MS4A1*, and *TCL1A*) (Matthias and Rolink, 2005) (Supplementary Figures 2A–C).

Upon over-clustering each sample, we observed a dominant proportion of immature thymocytes in week 9, marked by pre-TCR α chain (*PTCRA*), TCR δ chain (*TRDC*) and recombination activating gene 1 (*RAG1*) (Supplementary Figures 3A,B). We then subset immature populations from each sample and performed integration to characterize the global intrathymic differentiation of T cells. Putative dying populations that exhibited low gene detection rate (nGene) and high expression of stress-related genes (*DNAJA1*, *DNAJB1*, *DNAJB6*, and *HSP90AA1*) in weeks 11, 13, and 15 were discarded to avoid technical confounding caused by sample preparation (Denisenko et al., 2020) (Supplementary Figure 3C).

**FIGURE 2:**
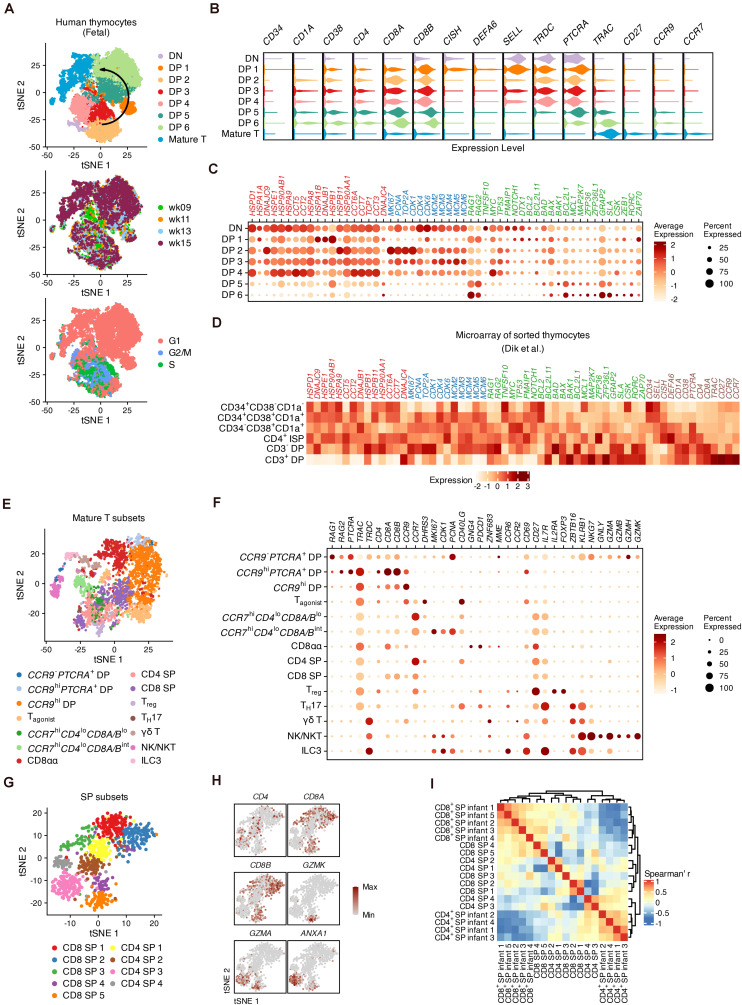
The global heterogeneity of differentiating human thymocytes. **(A)** Visualization of differentiating thymocytes with t-distributed stochastic neighbor embedding. (t-SNE) for cell populations (top), fetal stages (middle) and cell-cycle phases (bottom). **(B)** Violin plots for marker expression in each population with colors corresponding to panel **(A)**. **(C)** Dot plots for specific gene sets associated with heat shock proteins (red), the cell cycle (blue) and regulators of proliferation and differentiation (green). **(D)** Expression heatmap of genes corresponding to panels **(B,C)** in microarray data of sorted thymocytes. **(E)** Visualization of the heterogeneity in mature thymocytes with t-SNE. **(F)** Dot plots for feature gene expression within mature thymocyte populations. **(G)** Visualization of the heterogeneity in conventional SPs with t-SNE. **(H)** Visualization of feature gene expression in conventional SPs with t-SNE. **(I)** Heatmap of the Spearman correlation matrix of conventional SP subsets and sorted SPs measured with bulk RNA-seq.

Thymocyte populations were assigned based on the expression of well-known markers (Figures 2A,B and Supplementary Figures 4A,B). The transition from DNs to DPs was marked by upregulation of *CD4*, *CD8A*, *CD8B*, *CD38*, and *CD1A* expression (Terstappen et al., 1992). *CD34* was barely detected in the DN cluster indicating that most DNs we captured were differentiated hematopoietic stem cells (HSCs) (Haddad et al., 2006). DP 1 cells were enriched for *DEFA6* and *CISH*, two markers of the CD4 immature single positive (CD4 ISP) stage (Park et al., 2020). *SELL* (encoding CD62L), which has been reported as a well-known marker gene for mouse DNs (Bacon et al., 2018), was highly expressed in DN and DP 1 cells and was accompanied by *TRDC*. Maturation of differentiated thymocytes was characterized by increased expression of TCR-α constant gene (*TRAC*), TCR activation (*CD27*) as well as chemokine receptor genes (*CCR9* and *CCR7*) associated with the migration of T cell precursors (Taghon and Rothenberg, 2008; Halkias et al., 2014). Differentially expressed genes (DEGs) across all stages were verified with published microarray data (Dik et al., 2005) (Supplementary Figures 4C,D).

Fifty-four previously reported candidate genes related to thymocyte proliferation, elimination and TCR recombination were then investigated (Figures 2C,D). Proliferation in DP 2–4 cells was marked by upregulation of cell cycle markers such as *MKI67*, *PCNA*, and genes encoding cyclin-dependent kinases (CDKs) and mini-chromosome maintenance (MCM) proteins. Notably, a set of genes encoding heat shock proteins (HSPs) were highly expressed within DNs and early DPs, but were downregulated in late DPs, in both our scRNA-seq data and previously published microarray data (Dik et al., 2005). With the decrease of cell-cycle genes, the expression of TCR recombination genes (*RAG1* and *RAG2*) was increased in DP 5–6 cells. As potential regulators, we focused on B cell lymphoma 2 (BCL2) family genes during this process (Hernandez et al., 2010). *BCL2* and *PMAIP1* were upregulated in DNs and early DPs, together with conventional regulators of proliferation and apoptosis such as *MYC* and *TP53*, while *BCL2L11*, *BAD*, *BAX*, *BAK1*, and *MCL1* gradually increased during the maturation of DPs. *MAP2K7*, which is related to the p38 MAP kinase pathways involved in the elimination of DPs (Rincón et al., 1998), was also highly expressed in a small proportion of DP 5–6 cells. Additionally, Src and Syk family kinase genes (*GRAP2*, *SLA*, and *CSK*) were upregulated in late DPs; these proteins are known to be critical for the positive selection of mouse thymocytes (Starr et al., 2003).

We subset the mature T populations in Figure 2A and performed iterative clustering to examine the refined heterogeneity. Fourteen sub-clusters were assigned according to the expression of markers encoding canonical surface antigens or chemokine receptors (Figure 2E). In addition to conventional late DPs (*CCR9*^–^ or *CCR9*^hi^) (Halkias et al., 2014), *GNG4*^+^ CD8αα T cells (Verstichel et al., 2017), CD4 or CD8 SPs and *FOXP3*^+^*IL2RA*^+^ regulatory T cells (T_reg_) (Savage et al., 2020), our data also identified non-conventional lymphocytes including γδ T cells expressing *ZBTB16* and *KRLB1*, T_H_17-like cells expressing *CD40LG* and *CCR6* (Wang et al., 2009), innate lymphoid cells (ILC3s) expressing *IL7R* as well as natural killers (NK/NKTs) expressing *NKG7*, *GNLY* and granzyme genes (Engel et al., 2016; Yang et al., 2018; Figures 2F and Supplementary Figures 5A–C).

We then identified potential subpopulations of conventional SPs (CD4 SPs and CD8 SPs in Figure 2E) whose DEGs were verified with published microarray data (Dik et al., 2005; Figure 2G and Supplementary Figures 6A,B). We observed that CD8 SP 5 and CD4 SP 3 cells highly expressed the granzyme gene *GZMA* as well as *ANXA1*, whose endogenous expression may play a role in regulating both the positive and negative selection of the TCR repertoire (Paschalidis et al., 2010; Figure 2H). In addition, CD8 SP 5 cells expressed well-known markers for cytotoxic T cells (*CCL5*, *GZMK*, and *NKG7*) (Supplementary Figure 6A). Gene ontology (GO) analysis showed that these two subpopulations were enriched for genes related to adaptive immunity compared to other subpopulations, suggesting their functional maturation (Supplementary Figure 6C). After calculating the Spearman correlations between the scRNA-seq and bulk RNA-seq data, hierarchical clustering suggested that CD8 SP 4–5 and CD4 SP 3–4 cells were most similar to the sorted SP thymocytes (Helgeland et al., 2020; Figure 2I). Thus, our analysis profiled the global intrathymic differentiation of T cells in human fetuses.

### Common Features Modeling the Bifurcation of TCR Decisions Across Fetal Development

**FIGURE 3:**
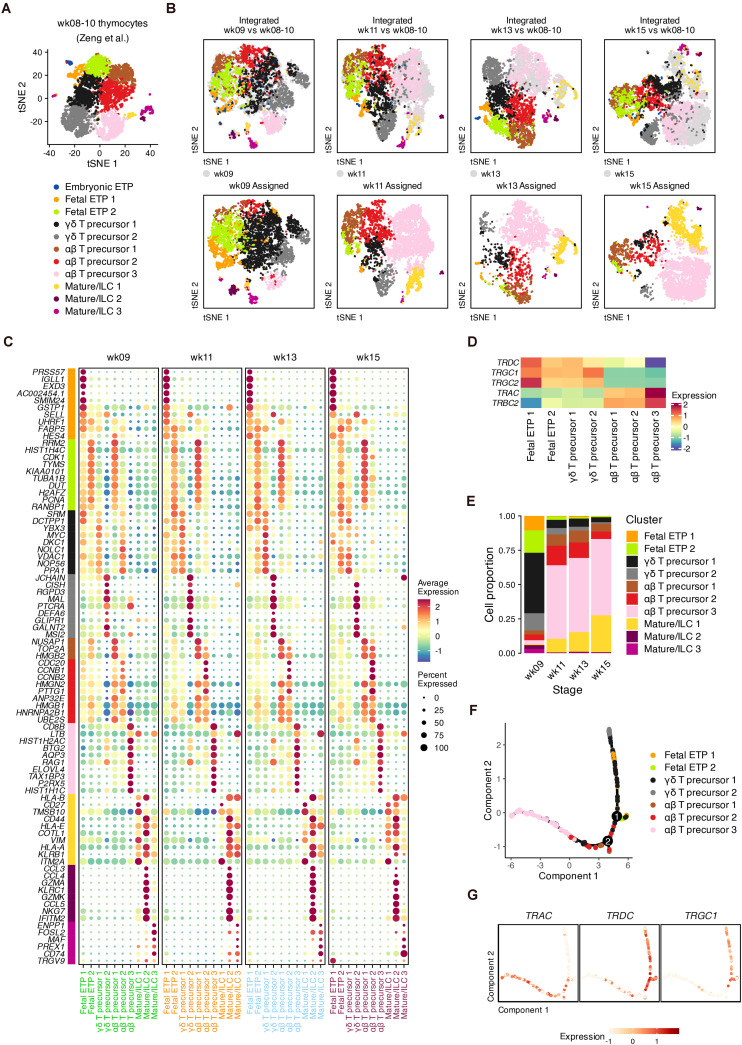
Conserved signature associated with lineages of T cell precursors during fetal development. **(A)** Visualization of thymocyte populations in the early human thymus (week 8–10). **(B)** Label transfer from the early human thymus to each developmental stage. Top: Coembedding of cells from the query and reference data. Bottom: Visualization of transferred labels in each developmental stage. **(C)** Dot plots showing conserved DEGs for transferring cell identities with colored text corresponding to developmental stages. **(D)** Expression heatmap of TCR genes for transferring cell identities. **(E)** Proportion of each transferred cell identities in each developmental stage. **(F)** Trajectory of the αβ and γδ lineages for pooled thymocytes, inferred using conserved DEGs in panel **(C)**. **(G)** Expression of TCR genes in the constructed trajectory in panel **(F)**.

We next investigated TCR gene expression decisions across fetal stages, which were masked by the integration mentioned above (Supplementary Figures 3A,B, 4A,B). Published data from the early fetal thymus (weeks 8–10) were reanalyzed as reference data (Figure 3A and Supplementary Figures 7A–E), and putative αβ and γδ precursors were clearly distinguished by TCR genes (Zeng et al., 2019) (Supplementary Figure 7E). We then used the reference to map and transfer the lineage information to our data (Figure 3B). We identified DEGs that discriminated cell states but were conserved across the four gestational ages (Figure 3C and Supplementary Table 1). These conserved DEGs were also verified within the original data from the early thymus (Supplementary Figure 7F).

In the two fetal ETP populations, the B cell lineage gene *IGLL1* and the neutrophil serine protease gene *PRSS57* were highly expressed by ETP 1 compared to ETP 2 cells, suggesting the further T cell lineage commitment of ETP 2 cells (Figure 3C). Compared to γδ precursor 1 cells, αβ precursor 1–2 cells at each gestational age highly expressed cell cycle genes such as *TOP2A*, *CDC20*, and high mobility group-box (HMGB) superfamily genes. Of the DEGs related to γδ precursor 2 cells, we found that *DEFA6* and *CISH* was upregulated, indicating an overlap with CD4 ISPs (Figure 3C and Supplementary Figure 7D). *MAL*, which encodes a T cell differentiation protein, and the transcription factor *HIVEP3*, which is related to TCR-dependent selection, were also enriched in γδ T precursor 2 cells (Figure 3C and Supplementary Figures 8A,B). Other γδ-precursor-related genes we detected such as *RGPD3*, *GLIPR1*, and *GALNT2* have rarely been studied regarding their functions in T cell differentiation. For αβ precursor 3 cells, we identified several surface marker genes (*CD4*, *CD8B*, and *CD38*), transcription factor 7 (*TCF7*) and the cell cycle inhibitor *BTG2* (Figure 2C and Supplementary Figures 8A,B). Other DEGs in αβ precursor 3 cells included *AQP3*, *SMPD3*, and *ELOVL4*, which have been reported in other scRNA-seq datasets as markers of late DPs (Park et al., 2020).

The segregation of TCR genes within our data was captured by the transferred annotations (Figure 3D). In week 9, the dominant population consisted of γδ T precursors, which were mostly replaced by αβ T precursors at week 11 (Figure 3E). This result was consistent with the high expression of *TRDC* at week 9 (Supplementary Figure 3B).

To confirm whether the conserved DEGs represent the distinction of the αβ-TCR and γδ-TCR lineages, we performed pseudotime analysis (Qiu et al., 2017). After removing cell cycle genes and non-variable genes, the remaining conserved DEGs were input to infer the differentiation trajectory (Figure 3F). The two main lineage bifurcations were clearly discriminated by *TRAC* as well as by *TRDC* and *TRGC1* in low-dimensional space (Figure 3G). Therefore, the conserved DEGs we identified can be considered potentially associated with the transcriptional alterations of different TCR genes regardless of the developmental stage.

### Lineage Commitment and Protein Maturation During Fetal Development

**FIGURE 4:**
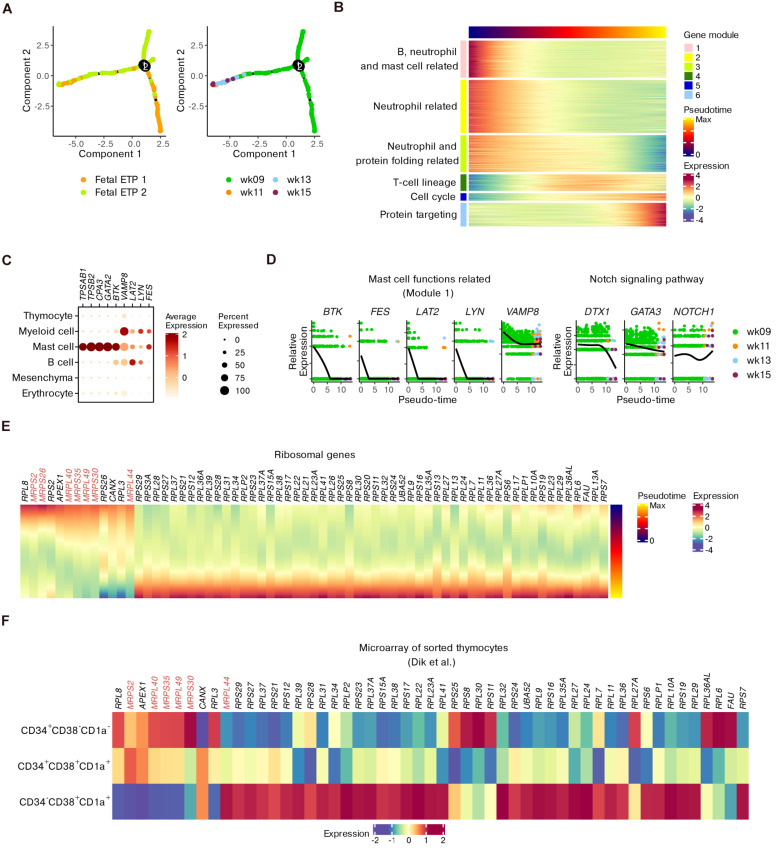
Refined trajectory of ETP differentiation during fetal development. **(A)** Trajectory of ETPs ordered using differential genes across fetal stages. **(B)** Heatmap showing gene modules altered across pseudotime scores computed in panel **(A)**. **(C)** Dot plots for the expression of representative genes corresponding to mast cell functions within the whole dataset. **(D)** Expression of genes in panel **(C)** as well as *DTX1*, *GATA3*, and *NOTCH1* across pseudotime scores. **(E)** Heatmap of ribosomal genes altered across pseudotime scores. MRP genes are highlighted. **(F)** Heatmap showing the expression of ribosomal genes identified in panel **(E)** within microarray data of sorted human thymocytes.

Although conserved features of T cell precursors were detected, we wondered how the transcriptional profile could be perturbed while being involved in fetal development. We subset ETPs since they represent the most important stage of T cell commitment (Mingueneau et al., 2013). The trajectory for ETP differentiation was inferred by setting DEGs across the four gestational weeks as “ordering genes” to model the potential effects of fetal development (Figure 4A).

Six gene modules that were dynamically altered along with pseudotime were identified (Figure 4B and Supplementary Table 3). GO analysis showed that early fetal ETPs were enriched for genes associated with the functions of B cells, neutrophils and mast cells (modules 1–3) and with protein folding (part of module 3). However, late fetal ETPs predominantly expressed genes related to T cell selection and differentiation (modules 4 and 5) and protein targeting to the endoplasmic reticulum (ER; module 6) (Figure 4B and Supplementary Figure 9A).

We investigated the kinetics of genes related to mast cell functions including *BTK*, *VAMP8*, *LAT2*, *LYN*, and *FES* (gene module 1). We confirmed the enrichment of these five genes within non-T cell populations compared to thymocytes (Figure 4C). In ETPs, the expression of these genes decreased from week 9 to week 15 (Figure 4D), even though a few ETP 1 cells were still present at the end of pseudotime (Figure 4A). We observed downregulation of the Notch signaling genes *DTX1* and *GATA3*, whose overexpression may induce ETPs to move toward the mast cell lineage (Taghon et al., 2007). However, *NOTCH1* itself did not show an obvious decrease (Figure 4D). Additionally, genes associated with other non-T cell lineages were also downregulated along with pseudotime (Supplementary Figure 9B). Therefore, we characterized the non-T cell potentials of ETPs and found that ETPs committed toward the T cell lineage during fetal maturation.

We further focused on the expression variations in ribosomal genes during ETP development, inspired by previous mouse data. Some genes encoding mitochondrial ribosomal proteins (MRPs) were dominantly expressed at an early stage while genes for conventional L and S ribosomal proteins (RPL and RPS) were upregulated at a later stage (Figure 4E). The transcriptional alterations were also confirmed by microarray data, especially for CD34^–^ DNs (Figure 4F). Together with the GO terms related to protein targeting, the upregulation of conventional ribosomal protein genes in late ETPs indicated that maturation of functional proteins occurred in these T precursors.

### Comparison of Thymocyte Proportions Between Humans and Mice

To compare the intrathymic T cell development between humans and mice, we used a similar analysis strategy on published scRNA-seq data from prenatal (E12.5-P0) and early postnatal (P6) mice and classified the DN, DP and mature populations (Bacon et al., 2018; Kernfeld et al., 2018) (Supplementary Figures 10A–F).

**FIGURE 5:**
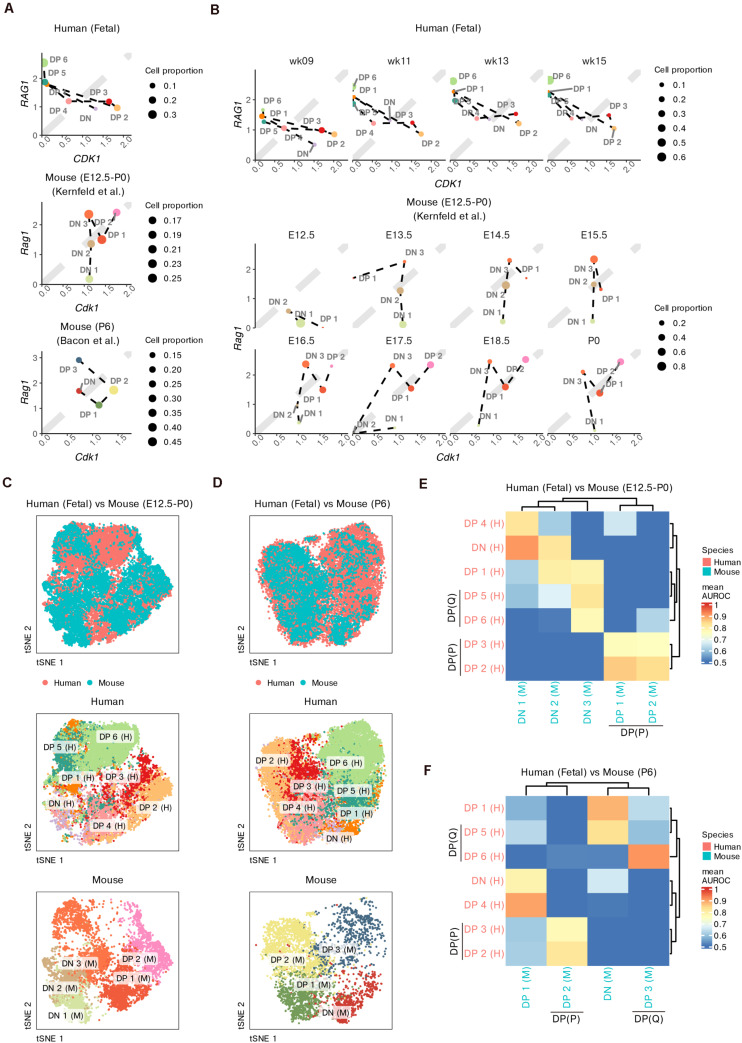
Integrative analysis of thymocyte differentiation in humans and mice. **(A)** Scatter plot comparing the expression of *CDK1* vs *RAG1* in thymocyte populations in humans and mice (prenatal and postnatal). **(B)** Scatter plot comparing the expression of *CDK1* vs *RAG1* within fetal thymocytes in humans and mice, divided by fetal stage. **(C,D)** Joint visualization of differentiating thymocytes in humans and mice. **(E,F)** Heatmap of area under the receiver operating characteristic (AUROC) curve scores between thymocyte populations from humans and mice based on the HVGs. P, proliferative; Q, quiescent.

First, we roughly compared the proportions of cells undergoing proliferation (marked by *CDK1*) and TCR recombination (marked by *RAG1*) among the three datasets (Figure 5A). All datasets contained proliferative DPs, namely, human DP 2–3, mouse prenatal DP 1–2 and mouse postnatal DP 2 cells. The simultaneous upregulation of *RAG1* and *CDK1* in mouse prenatal DP 1–2 cells suggested the transition between proliferation and TCR recombination. Non-proliferative DPs with upregulated *RAG1* and downregulated *CDK1* were not observed in the mouse fetal thymus. However, both human DP 5–6 and mouse postnatal DP 3 cells expressed low levels of *CDK1* but high levels of *RAG1*, suggesting cell quiescence and active TCR recombination in these DPs. Furthermore, there was already a small proportion of non-proliferative DPs in the human fetus as early as week 9, while the first wave of mouse non-proliferative DP did not appear until E16.5 (Figure 5B).

To better measure the conservation of thymocyte proportions between the two species, we integrated our data with the mouse data using homologous genes. Cells were co-embedded into a low-dimensional space. As expected, for thymocytes from both pre- and postnatal mice, DN populations roughly overlapped with human DN and DP 1 cells, while proliferative DPs overlapped well with human DP 2–3 cells (Figures 5C,D). Compared to mouse neonates, mouse fetuses contained barely any thymocytes that overlapped with human DP 6 cells, even though some cells from the mouse DN 3 population showed partial overlap with human DP 5 cells (Figure 5C).

We further used MetaNeighbor to measure the cellular similarity between the two species (Crow et al., 2018). Consistent with the integration results, DPs from prenatal mice were well matched only with proliferative DP 2–3 cells from humans (Figure 5E), while both the proliferative DP 2 and non-proliferative DP 3 cells from mouse neonates had similar populations to those from humans (Figure 5F). We also noticed that as early as week 9, human thymocytes already exhibited obvious similarities to mouse neonate thymocytes (Supplementary Figures 11A–D). The DN populations from postnatal mice were most similar to human DP 1 and DP 5 cells instead of human DNs (Figure 5F). Mouse prenatal DN 1–2 cells showed similarities with human DNs and partial DPs, while mouse prenatal DN 3 cells were most similar to human DPs (Figure 5E).

All these results suggest that there is a relative delay during the intrauterine development of mouse DPs compared with that of human thymocytes, but these differences become smaller during early postnatal stages. The DN populations may contribute to the cross-species differences in developing thymocytes.

### Conserved and Specific Gene Expression of Thymocytes in Humans and Mice

**FIGURE 6:**
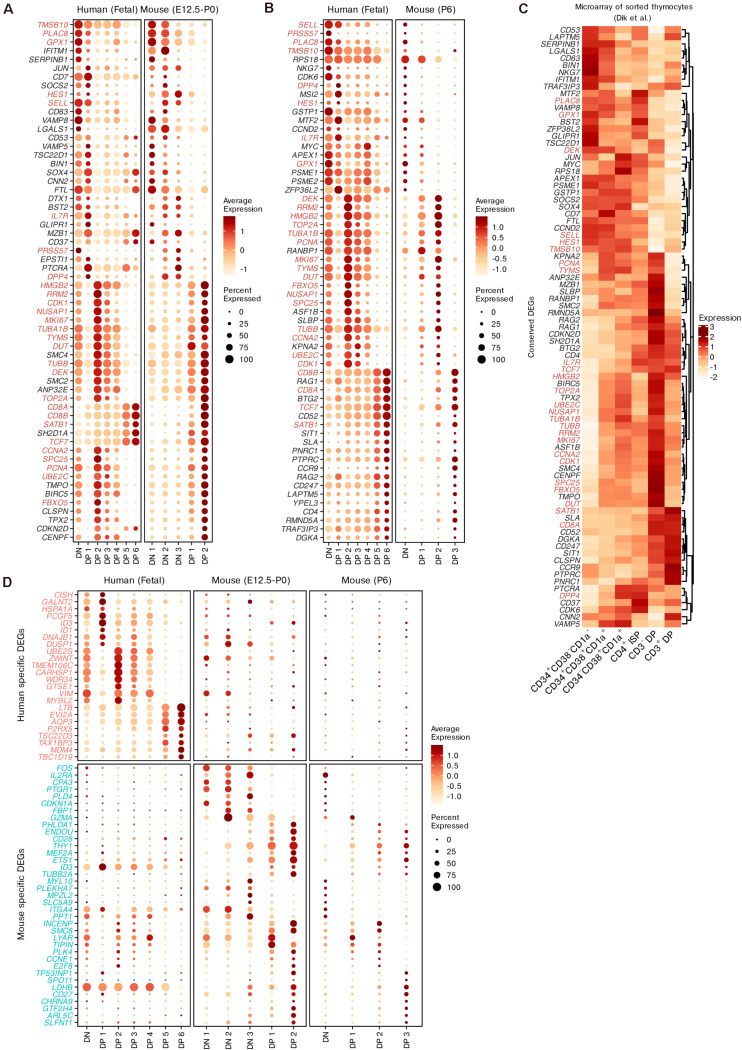
Conserved and species-specific features of thymocyte differentiation in humans and mice. **(A,B)** Dot plots showing conserved DEGs of differentiating thymocytes in humans and mice. The DEGs shared between pre- and postnatal are highlighted. **(C)** Expression of conserved DEGs corresponding to panels **(A,B)** within sorted human thymocytes. **(D)** Dot plots showing putative species-specific DEGs of differentiating thymocytes in humans and mice.

For both humans and mice, we identified the conserved DEGs while modeling differentiation from DNs to DPs using the integration method mentioned above (Supplementary Table 4). Although we found that the DN populations from both species had limited similarity, conserved markers that were not specific to T cell lineages were identified, such as *PRSS57* and *NKG7*. The expression of Notch pathway genes (*DTX1* and *HES1*) was also conserved in DNs and early DPs between the two species (Figures 6A,B). In differentiating DPs, the conserved DEGs included not only common surface markers (*PTPRC*, *IL7R*, *CD4*, *CD8A*, *CD8B*, and *CCR9*) but also regulators of thymic selection (*RORC*, *CAMK4* and *NFATC3*, see Supplementary Table 4) (Schmidt et al., 1998; Raman et al., 2001; Canté-Barrett et al., 2007), *TRAC* rearrangement (*RAG1, RAG2* and *SATB1*) and TCR signaling (*SKAP1*, see Supplementary Table 4) (Clements et al., 1999; Liu et al., 2008; Hao et al., 2015). We also identified many cell-cycle genes as conserved DEGs between the two species, including *TUBA1B*, *MKI67*, and *TOP2A* (Figures 6A,B). The conservation of gene expression was further confirmed by microarray data from sorted human thymocytes (Dik et al., 2005; Figure 6C).

Next, we identified subsets of potential species-specific DEGs among all homologous genes. The expression of the top species-specific DEGs was profiled (Figure 6D). *IL2RA* (encoding CD25) was highly expressed by mouse DNs as a conventional marker (Mingueneau et al., 2013), but was minimally expressed in human DNs. Furthermore, mouse DNs exhibited upregulation of some marker genes related to non-T lineages such as the mast cell marker *CPA3* and the granzyme gene *GZMA*. *ID3* was identified as a specific DEG in both species. In mice, this gene was upregulated in differentiating DPs. However, it was downregulated in human late DPs but upregulated in human DP 1 cells, which may represent the transition stage between DNs and DPs. The expression trends of *CD27*, *EVI2A*, *AQP3*, and *LDHB* were also different between the two species. Some DEGs exhibited similar expression trends but different detection rates between humans and mice, such as the cell-stress gene *FOS* and DP maturation markers (*CD28* and *LTB*). These results display the conserved and species-specific transcriptional dynamics of thymocyte differentiation between humans and mice.

### Specific Cell States Expressing Genes Associated With Autoimmune Disorders in GWAS

**FIGURE 7:**
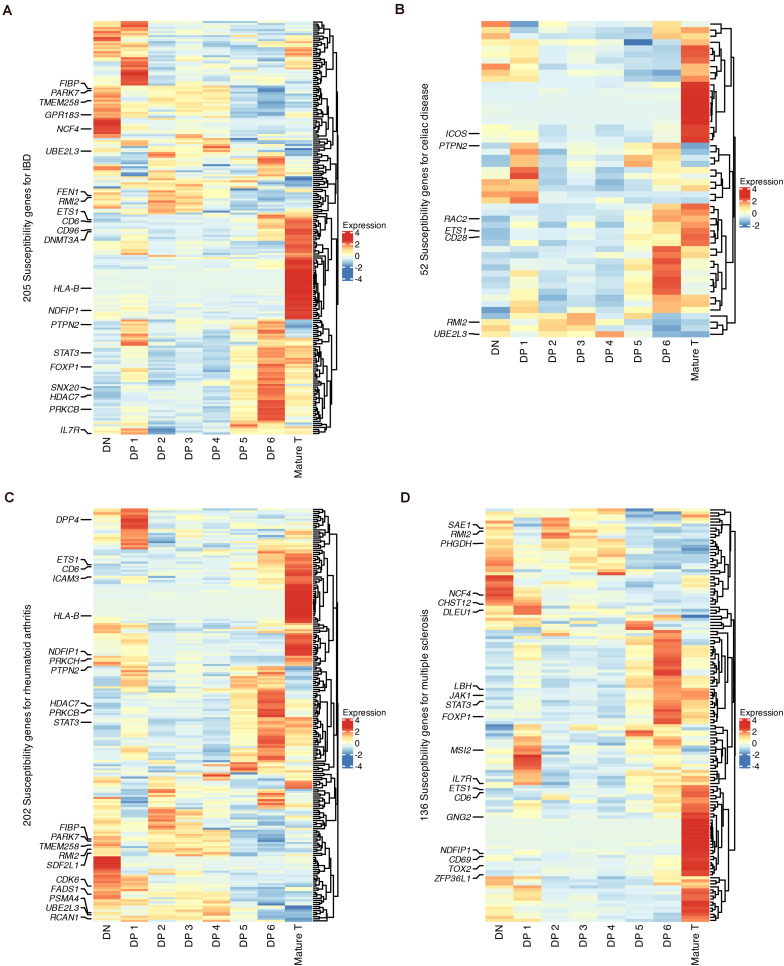
Expression of GWAS risk genes for human autoimmune disorders in developing thymocytes. **(A)** Heatmap of 205 susceptibility genes for IBD among differentiating thymocytes. **(B)** Heatmap of 52 susceptibility genes for celiac disease among differentiating thymocytes. **(C)** Heatmap of 202 susceptibility genes for rheumatoid arthritis among differentiating thymocytes. **(D)** Heatmap of 136 susceptibility genes for MS among differentiating thymocytes.

Finally, we used our data to explore the links between T cell development and autoimmune disorders, including inflammatory bowel disease (IBD), celiac disease, rheumatoid arthritis and multiple sclerosis (MS), which have been studied with previous mouse scRNA-seq data (Kernfeld et al., 2018). The cell-state enrichment of GWAS genes associated with these four diseases was profiled (Figures 7A–D). Although the numbers of susceptibility genes were different for these diseases, clustering analysis showed that these genes were consistently enriched in DN, DP 1, DP 6, and mature T populations. The cell-type enrichment of susceptibility genes was also verified using microarray data (Supplementary Figures 12A–D).

We highlighted GWAS genes that were differentially expressed in each state (Figures 7A–D). Some genes were shared by more than one disease, such as *NCF4*, *RMI2*, and *TMEM258* in DNs and *STAT3*, *HDAC7*, and *FOXP1* in DP 6 cells. Several known markers for T cell differentiation were also highlighted such as *TOX2*, *CD69*, and *CD28* (Taghon and Rothenberg, 2008). These results revealed that stages such as the DN, late DP and mature SP stages are genetically associated with autoimmune disorders.

## Discussion

By characterizing thymocyte differentiation during fetal growth at a single-cell resolution, our work fills a few neglected gaps regarding human fetal T cell development. We identified a set of conserved genes that contributed to TCR lineage decisions regardless of the developmental stage. We also mapped the trajectory of fetal ETP commitment toward T cell lineages and functional protein maturation across different gestational ages. The cross-species comparison of DNs and DPs reveals potential limitations of using murine models to study thymocyte differentiation. By profiling the expression of susceptibility genes, we have connected the autoimmune disorders with specific phases of human thymocyte development.

Thymocyte differentiation and fetal growth are two key issues that should be considered in the study of human T cell development. In mice, the gradual differentiation from DNs to DPs exhibits remarkable synchronicity with embryonic days (Kernfeld et al., 2018). However, the complete phases of DNs and DPs is detectable in the human thymus as early as week 9. At week 11, the proportion of thymocytes expressing *TRDC* greatly decreases, consistent with the fact that the γδ lineage dominates T lymphocyte immunity in the early fetus (Carding et al., 1990). These observations suggest that thymocyte differentiation and fetal growth are two different but synergetic biological processes beginning in the second trimester in humans. For single-cell data, comparison of the transcriptomic profiles between developmental time points is still challenging due to the inherent technical noise during data generation (Svensson et al., 2017). Previous scRNA-seq researches on thymic development usually integrated data from different gestational ages and then analyzed them as a combined dataset to identify common cell clusters (Zeng et al., 2019; Park et al., 2020). Our work provides some additional trials to investigate the influence of gestational ages when dealing with cell differentiation on scRNA-seq, using analysis strategies that are similar to other studies on human development (Li et al., 2017; Fan et al., 2018; Gao et al., 2018; Wang et al., 2018; Zhong et al., 2018; Cui et al., 2019).

With regard to the effects of gestational ages on the trajectory of fetal ETPs, we found that ETPs from the later-stage fetus (weeks 11–15) are more specific to T cell lineage. T cell lineage commitment results from the complicated interaction between HSCs and the thymic stroma, such as the epithelium (Han et al., 2020; Lavaert et al., 2020; Le et al., 2020). Since thymus organogenesis and the earliest seeding of HSCs begin almost simultaneously around week 8 (Farley et al., 2013), we can hypothesize that the T cell lineage specification of ETPs at later gestational ages may correspond to the gradual maturation of the thymic micro-environment during fetal growth. Taking together, our work illustrates the transcriptomic dynamic profile during the change of gestational ages. As for the limitation of our present study, the lack of enough biological replicates should be noticed. In fact, it is extremely difficult to accomplish the cross-age trajectory inference with necessary biological replicates due to limited clinical samples matching the criteria, time constraints and budgets. Alternatively, biological replicates that match a given criteria can be collected on different days. However, the potential batch effects may lead to confusing results (Bacher and Kendziorski, 2016). Fortunately, new bioinformatics tools that can simultaneously handle the cell-type identification, different time points and batch correction may help to improve the comparison of transcriptomic dynamic between conditions such as developmental time points (Crowell et al., 2020).

Although the bifurcation of αβ and γδ lineages has been studied in the human thymus from weeks 8–10 (Zeng et al., 2019), our data expand the gestational ages to the second trimester. Conserved feature genes that we identified in our data can guide further investigations into potential essential regulators of αβ- or γδ-TCR lineage decisions. For example, the transcription factor *HIVEP3*, which was enriched in human γδ T precursor 2 cells in our data was also observed to be highly expressed in mouse early γδ-T cells in a previous scRNA-seq study (Sagar et al., 2020). In our data, the putative γδ T precursors could also represent CD4 ISP thymocytes which have just finished the recombination of γδ TCR before αβ TCR (Taghon and Rothenberg, 2008; Park et al., 2020), and hence, annotating the lineages of T cell precursors simply based on *TRAC* and *TRDC* is not absolutely precise. Based on that, it is reasonable that directly sequencing the productively recombinant TCR genes will provide more precise understanding of lineage decision dynamics.

Heat shock proteins, which play indispensable roles in protein folding and the maintenance of genome integrity, have been reported to be involved in complicated cellular processes such as mitochondrial homeostasis and survival (Young et al., 2003; Beere, 2005). MRPs are specific to the synthesis of mitochondrial proteins (Sylvester et al., 2004). The enrichment of these genes indicated the predominance of energy metabolism in early thymocytes. Along with ETP differentiation, the upregulation of conventional ribosomal protein genes suggested that the synthesis of functional proteins is increased, which is essential for stem cell differentiation (Sanchez et al., 2016). In mice with fetal growth restriction (FGR), the defects of specific ribosomal protein genes are mainly related to the delay of DP maturation (Bacon et al., 2018). Based on our results, similar FGR-susceptibility in humans may emerge in early ETPs. Thus, our work maps the transition from fundamental renewal to functional differentiation within human thymocytes.

In comparison with human thymocytes, we observed that mouse DPs undergo a relatively delayed intrauterine development and become dominated by TCR-recombination after birth. The postnatal development of mouse thymocytes undisputedly reflects that Carnegie staging is not applicable for human fetuses since the second trimester (Farley et al., 2013), which reminds researchers of the reliability of using murine models. In humans, non-proliferative DPs largely emerge during development in the relatively sterile uterus. However, in mice, similar DP populations arise in the extrauterine environment, which is filled with antigens. Hence, intrauterine factors that induce the complete differentiation of human thymocytes in the second trimester require further exploration.

Cross-species comparisons of the differentiation from DP to SP thymocytes have been conducted before (Chopp et al., 2020; Park et al., 2020). However, our analyses suggest that the heterogeneity between the two species is partly caused by DN thymocytes, whose transcriptomic dynamics during differentiation toward DP cells have not been compared on previous studies. Although the key features such as T cell lineage commitment, Notch signaling genes, proliferation and TCR rearrangement were conserved, we still identified the species-specific transcriptomic features of DN and DP thymocytes. The transcriptomic differences between humans and mice are not only influenced by the expression trends of well-known functional genes across cell types, but also by the cell proportions with upregulation of cell-type marker genes. For example, *ID3*, a transcription factor that promotes the γδ T cell fate (Lauritsen et al., 2009), are highly expressed by different thymocyte populations in humans and mice, which indicates the potential species-specific bifurcation points of TCR lineages. In short, our discoveries provide important insights for future cellular studies to investigate the thymic development of human T cells using murine thymocytes.

Immune disorders, including immune deficiency and autoimmunity, are reported to arise from the abnormal development of T cells (Cavadini et al., 2005). Recently, a cell atlas study explored the relationship between severe combined immunodeficiency (SCID) and cellular heterogeneity in the developing thymus (Park et al., 2020). Notably, GWAS genes located near single-nucleotide polymorphisms (SNPs) associated with human autoimmune diseases are enriched in non-conventional T cells during murine thymic development (Kernfeld et al., 2018). These GWAS genes also showed cell-state specific enrichment in our data, indicating their potential functions in several phases during normal thymocyte differentiation, such as T lineage commitment, TCR recombination and SP maturation. Genetic mutations in these phases could be related to the susceptibility of autoimmune disorders such as IBD, MS, celiac disease and rheumatoid arthritis. Therefore, future studies on the mechanisms underlying multiple immune disorders should take specific thymocyte states during embryogenesis into account.

In summary, our scRNA-seq map and integrative analysis allow us to compare features across datasets and species. Our results provide an improved multiple-layer profile of human fetal thymic development and organogenesis, and reconstruct a substantial molecular framework to complement the baseline in the thymus and immune disorders. Importantly, the intrathymic differentiation of T lymphocytes at the intrauterine stage is more complicated than previously thought, and integrative analysis with more datasets from single-cell multi-omics will be necessary to obtain more comprehensive insights of human T cell development in the future.

## Materials and Methods

### Human Sample Collection

Human fetuses were collected from healthy donors at gestational weeks 9–15 following the elective medical termination of pregnancy at the International Peace Maternity and Child Health Hospital (IPMCH). Donors voluntarily provided written informed consent, and no financial inducements were offered for donation. The collected fetuses were clinically confirmed to be free of any known genetic or developmental abnormalities. All protocols were reviewed and approved by the Ethics Committee of IPMCH (GKLW-2017-81). The developmental age was estimated by measuring the standard crown-rump length (CRL) with ultrasound (Papageorghiou et al., 2014).

### Thymocyte Isolation and scRNA-seq

All human fetuses were washed in cold PBS for 3–4 times, and then the thymus lobes were dissected in pre-cooled RPMI 1640 medium (Gibco, 11875093) containing 10% fetal bovine serum (FBS, Gibco, 10437-028). Cell suspension was prepared by gently smashing the thymus through a 70-μm cell strainer (Falcon, 352350). The suspension was treated with 1× red blood cell (RBC) lysis buffer (eBioscience, 00-4333-57) at 4°C for 5 min, and the remaining cell suspension was washed with PBS + 2% BSA. After filtering through a 35-μm cell strainer (Falcon, 352235), cells were stained with 7-AAD (BioLegend, 420403), and 7-AAD^–^ viable cells were sorted using a BD FACSAria III cell sorter and finally resuspended in PBS + 0.04% BSA at a concentration of 700–1,200 cells/μL.

Cell viability was measured by a trypan blue assay, and samples with more than 80% viability were used for library construction. Then scRNA-seq libraries were prepared using a Chromium Single Cell 3′ Reagent Kit (Version 2, 10× Genomics) (Zheng et al., 2017) following the manufacturer’s protocol. The cDNA libraries were sequenced on an Illumina HiSeq X Ten platform in the paired-end 150 bp mode.

### Public Datasets

Human early fetal thymus (8–10 weeks of gestation) datasets were obtained from Gene Expression Omnibus (GEO; GSE133341), in the FASTQ file format (Zeng et al., 2019). Mouse thymus datasets were obtained from GEO (GSE107910; Fetal, E12.5-P0) and ArrayExpress (E-MTAB-6945; Postnatal, P6) in the expression matrix format (Bacon et al., 2018; Kernfeld et al., 2018). Normalized microarray data from sorted human thymocytes were retrieved from GEO (GSE22601) using the GEOquery R package (version 2.54.1) (Dik et al., 2005). For bulk RNA-seq data of sorted human SP thymocytes, raw count data were directly downloaded from GEO (GSE139242) (Helgeland et al., 2020). All publicly available data on sample information, such as grouping labels and fetal stages, was referred to the descriptions in GEO.

### Data Preprocessing

All sequencing files were aligned and quantified with CellRanger Software (version 3.0.2, 10× Genomics), using the default parameters and the GRCh38 human reference genome (official Cell Ranger reference, version 1.2.0). Generated expression matrices were used for downstream analyses. Published bulk RNA-seq data were preprocessed with the DESeq2 R package (version 1.26.0) with the default parameters.

### Basic Analysis Pipeline for scRNA-seq Data

Most analyses were performed using the Seurat R package (version 3.1.1) (Stuart et al., 2019). Unless specially declaration, we employed SCTransform, a regularized negative binomial regression method to normalize and standardize the unique molecular identifier (UMI) count matrix, and the log10 transformed number of total UMIs per cell was used as an indicator of sequencing depth (Hafemeister and Satija, 2019). Highly variable genes (HVGs) were identified based on the variance of Pearson residuals. After the principal component analysis (PCA) of HVGs, a shared nearest neighbor (SNN) graph was generated using the top significant principal components (PCs) determined by an elbow plot. We then clustered cells into transcriptionally distinct populations based on the Leiden algorithm (Traag et al., 2019). The visualization of cell populations was performed with t-stochastic neighbor embedding (t-SNE).

Data generated with LogNormalize was used for visualization and differential expression (DE) analysis with the Wilcoxon rank-sum test. The p-values were adjusted for multiple testing with the Bonferroni correction. Genes with an adjusted *p*-value < 0.05 were defined as DEGs. Cell clusters were annotated according to DEGs and canonical cell-type markers. The GO analysis of DEGs was carried out with the ClusterProfiler R package (version 3.14.0) (Yu et al., 2012), and the cutoffs were set to 0.01 for the *p*-value and 0.05 for the q-value.

### Cell Cycle Analysis

The cell cycle score of each cell was calculated based on the expression of G2/M and S phase core marker genes (Tirosh et al., 2016). A linear model was implemented to regress cell cycle effects. We stored cell cycle phase information before regression, allowing us to evaluate changes in the proliferation state.

### Quality Control and Contaminated Cell Removal

When we initially clustered the cells, we found that some cells were contaminated by hemoglobin genes such as *HBA1*, *HBA2*, and *HBG2*, suggesting the existence of ambient mRNAs. Thus, we implemented the soupX R package (version 1.2.1) to remove contaminating mRNAs (Young and Behjati, 2020). Hemoglobin genes (*HBB*, *HBD*, *HBG1*, *HBG2*, *HBE1*, *HBZ*, *HBM*, *HBA2*, *HBA1*, and *HBQ1*) were set as “non-Expressed Gene List” to estimate contaminant fractions. The removed counts predominantly consisted of *MALAT1*, a lncRNA frequently detected in poly-A-captured RNA-seq (Islam et al., 2011), ribosomal genes and mitochondrial genes.

Doublets with gene profiles of multiple cell types can perturbate classification analysis. We merged the four thymus datasets, performed an initial clustering analysis, and roughly identified the main cell types, which consisted mostly of thymocytes. Cross-type doublets were detected with the DoubletDecon R package (version 1.13) (DePasquale et al., 2019). The expression of cell-type markers in doublets was also examined.

The final cleaned data included cells containing more than 500 detected genes, fewer than 40,000 UMI counts, more than 10% ribosomal genes (GO: 0005840) and less than 4% mitochondrial genes (Ilicic et al., 2016). Mitochondrial genes and genes that were expressed in fewer than three cells were removed from further analyses.

### Integration Analysis of Datasets From Different Batches

We implemented the newest Seurat method to identify the correspondences between cells from different datasets (Stuart et al., 2019). In each integration case, common variable genes were detected with the SelectIntegrationFeatures function. Then, cell pairwise “anchors” were identified using the FindIntegrationAnchors function. Batch-corrected matrices were generated with the IntegrateData function.

### Detection of Conserved DEGs

We used the FindConservedMarkers function to identify genes that had similar cell-type expression trends in each group (developmental age or species), i.e., conserved DEGs (Butler et al., 2018). Briefly, differential expression tests were run separately within each group, and the combined p-values were calculated with the minimump function in metap R package. Genes with a combined *p*-value < 0.05 were considered as conserved DEGs. We further retained genes with an adjusted *p*-value < 0.05 within each differential expression test.

### Label Transfer

Annotations of cell lineages in the early fetal thymus were re-assigned using the pipeline mentioned above with the original article as a reference. Then, we transferred these cell lineage labels to our data using the FindTransferAnchors and TransferData functions in Seurat, a strategy that is similar to data integration. Cells with a maximal prediction score < 0.5 were discarded. Integration and conserved DEG detection were then performed.

### Trajectory Analysis

The monocle R package (version 2.14) was used to investigate the conservation of αβ and γδ T lineage differentiation (Qiu et al., 2017). To obtain “ordering genes,” we used the intersection of conserved DEGs and common variable features identified at each time point, selected the top 50 upregulated and downregulated DEGs and removed the cell cycle genes (GO:0007049). The cell trajectory was computed based on “DDRTree” dimensionality reduction and the pseudotime score was assigned with the orderCells function.

To model transcriptional dynamics through fetal stages, we identified “ordering genes” from HVGs using the differentialGeneTest function in monocle. The fullModelFormulaStr parameter was set to “∼Stage.” Genes with q-values < 0.001 were retained. After the removal of cell cycle genes, we performed pseudotime analysis, as mentioned above. Genes altering as a function of pseudotime were detected with the differentialGeneTest function, with the cutoff q-value set to < 10^–7^. GO analysis for gene modules was carried out with ClusterProfiler. Ribosomal genes were obtained with the GO database (GO: 0005840).

### Spearman Correlation Analysis Between scRNA-seq and Bulk RNA-seq Data

We used the AverageExpression function in Seurat package to make the scRNA-seq data comparable to the bulk RNA-seq data. We then merged these two types of data as one expression matrix. A matrix of Spearman correlations (Spearman’s *r* values) across cell types was calculated to evaluate the transcriptomic similarities. A heatmap was drawn using ComplexHeatmap R package. Hierarchical clustering was used to cluster the most similar cell types.

### Comparison of Human and Mouse Data

Mouse datasets were reanalyzed with a basic pipeline, and cell states were labeled according to gene expression profiles and the original articles. For cross-species integration, mouse genes were converted into human homologous genes using the biomaRt R package (version 2.42.1). Genes with mutually unique conversions were retained. Following integration across species, subtypes of thymocytes were merged into two main differentiation stages (DN and DP). Cell-cell similarity across species was computed on the integrated matrix using the MetaNeighbor R package (version 1.6.0) (Crow et al., 2018). We then detected conserved DEGs as mentioned above.

### Susceptibility Genes for Autoimmune Disorders Identified Through GWAS Data

Susceptibility SNPs of IBD, MS, celiac disease and rheumatoid arthritis were downloaded from the GWAS catalog after searching by keywords (Buniello et al., 2019). SNPs with a *p*-value < 5 × 10^–8^ were retained. Candidate genes mapped by the remaining SNPs were obtained to analyze the relationships between their expression and human thymocyte development.

## Data Availability Statement

The datasets presented in this study can be found in online repositories. The names of the repository/repositories and accession number(s) can be found below: https://www.biosino.org/node/project/detail/OEP001185.

## Ethics Statement

The studies involving human participants were reviewed and approved by the Ethics Committee of the International Peace Maternity and Child Health Hospital (GKLW-2017-81). The patients/participants provided their written informed consent to participate in this study.

## Author Contributions

XML conceived the idea and administrated the project. YCL, YYG, and LLB collected the tissue samples with help from XYH, GLD, and LJ. YCL, TL, and YYG conducted the experiments. GYZ and YCL analyzed the data. YCL and WHZ drafted the first version of the manuscript. WHZ and XML reviewed the manuscript. All authors reviewed the draft for intellectual content and approved submission of the final version of the manuscript.

## Conflict of Interest

The authors declare that the research was conducted in the absence of any commercial or financial relationships that could be construed as a potential conflict of interest.
