# Supplementary figures and images for: Integrative Single-Cell Transcriptomic Analysis of Human Fetal Thymocyte Development
Source: Front Genet. 2021 Jul 2;12:679616. doi: 10.3389/fgene.2021.679616 (PMC8284395; doi:10.3389/fgene.2021.679616)

**A**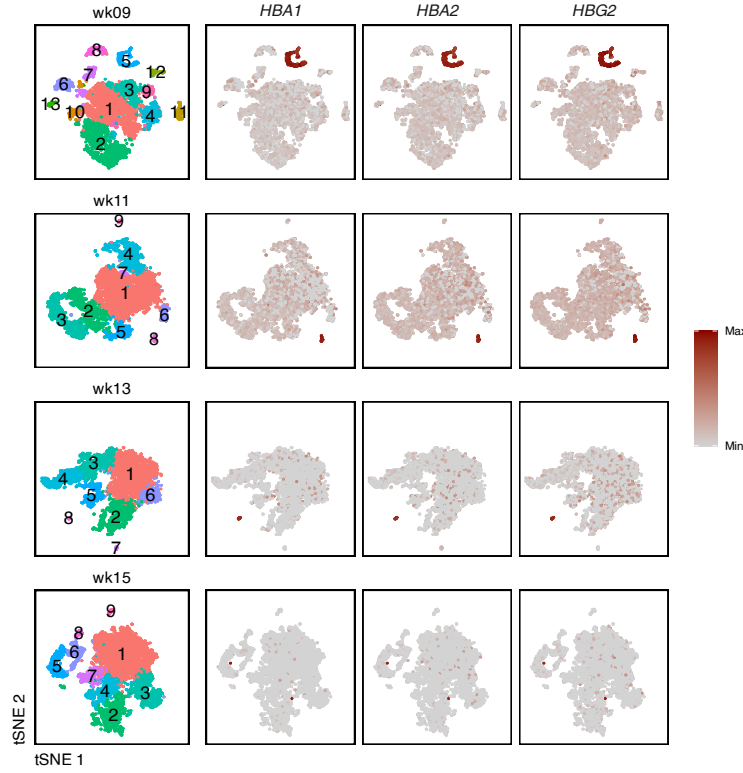**B**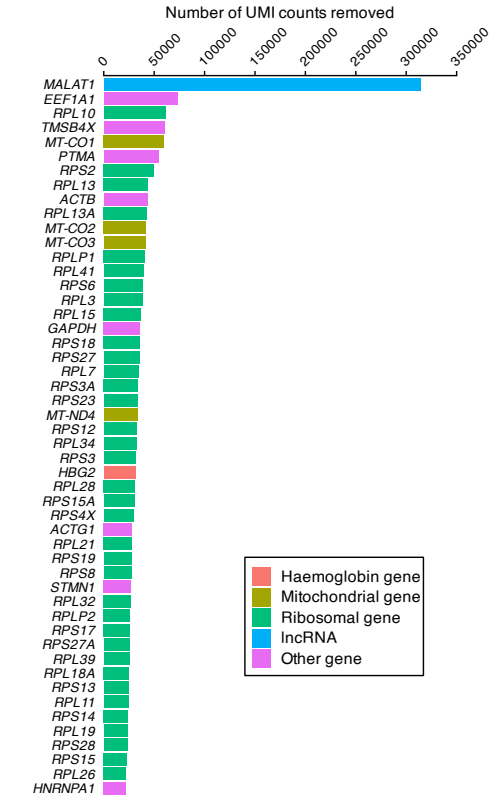**C**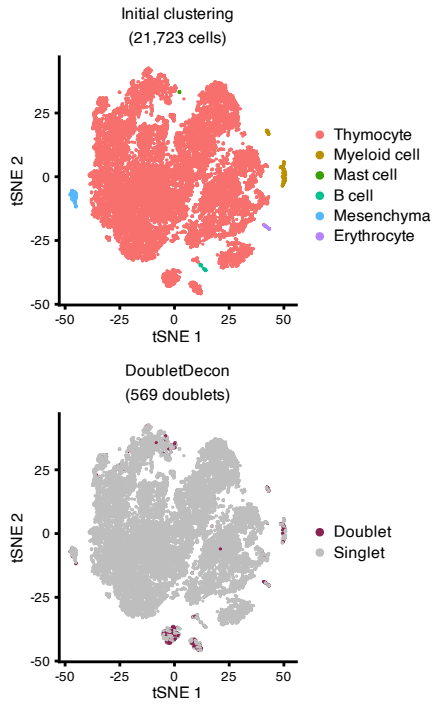**D**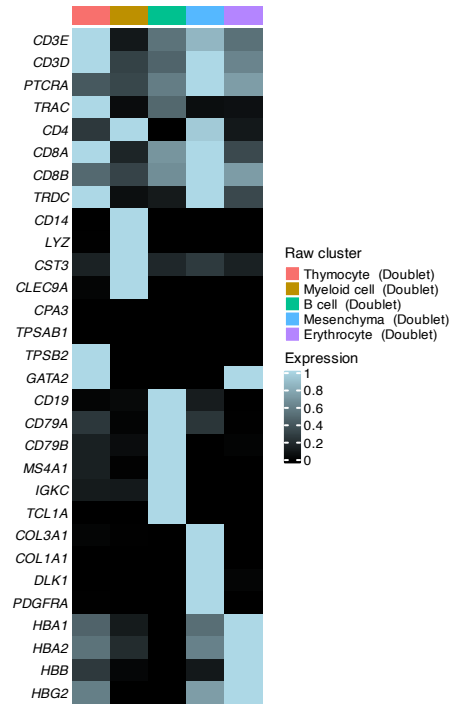**E**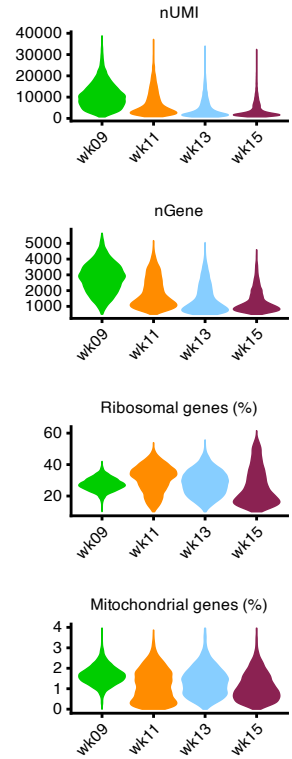

Supplement: Supplementary file 5 [file Data_Sheet_1.PDF]

**A**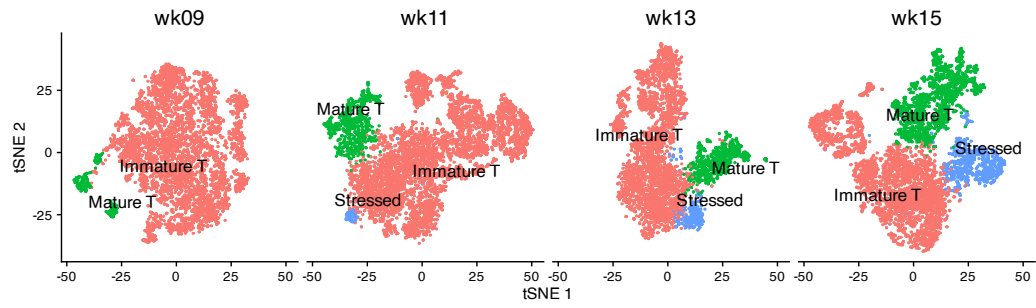**B**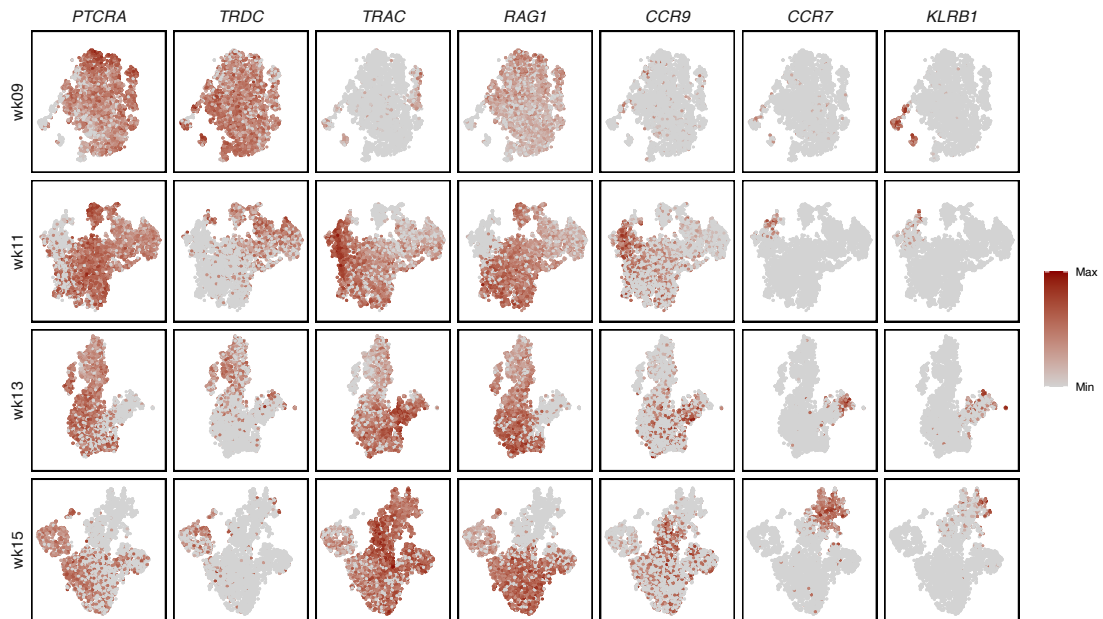**C**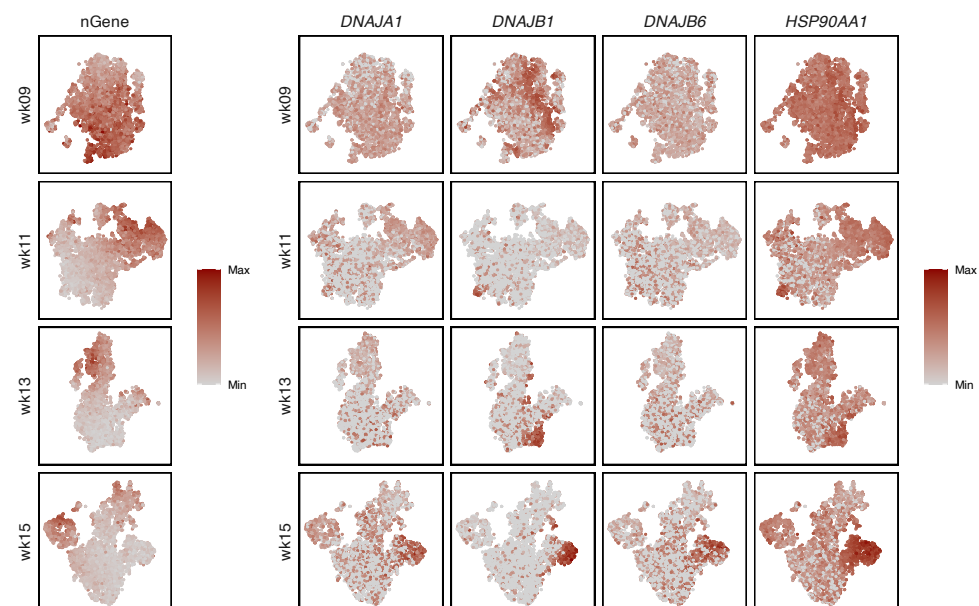

Supplement: Supplementary file 7 [file Data_Sheet_3.PDF]

A

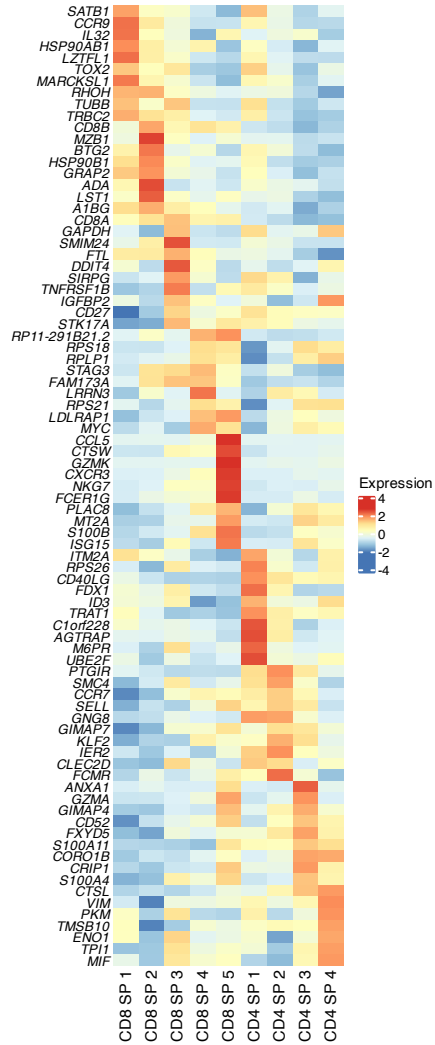

B

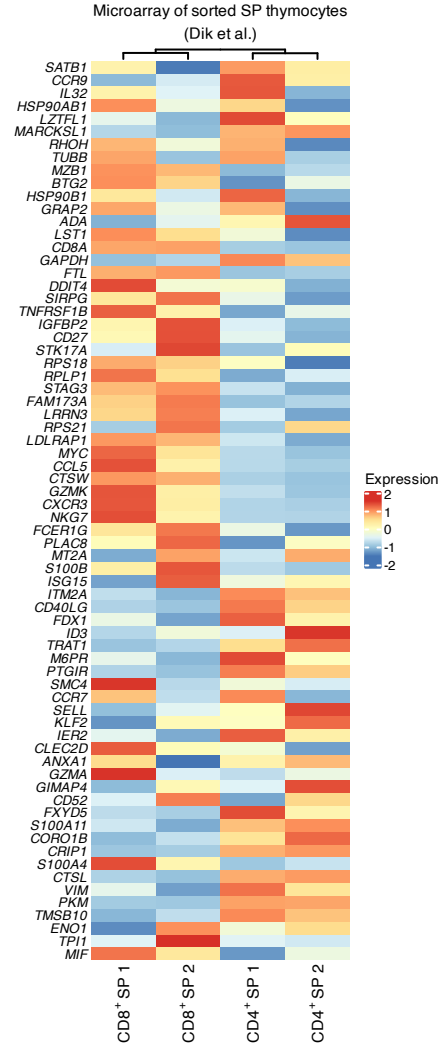

C

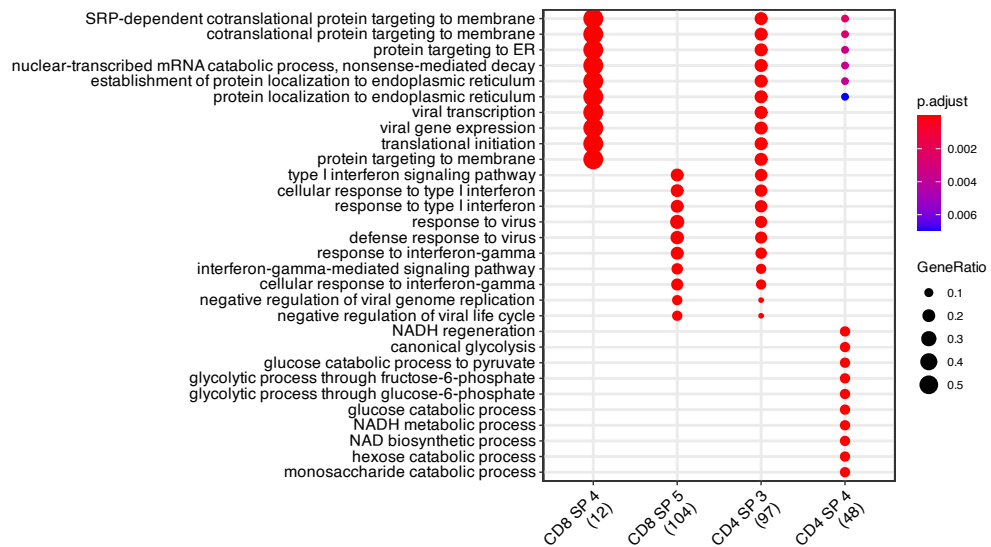

Supplement: Supplementary file 10 [file Data_Sheet_6.PDF]

A

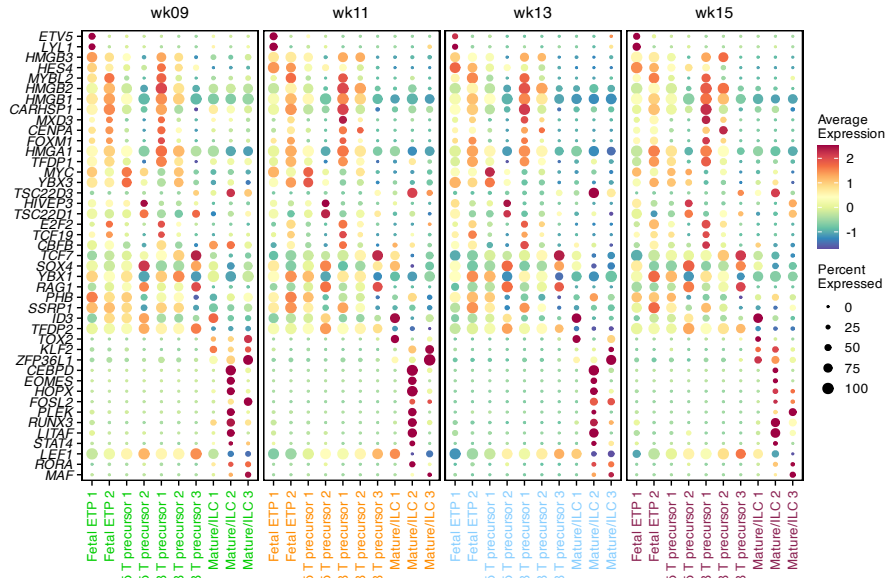

B

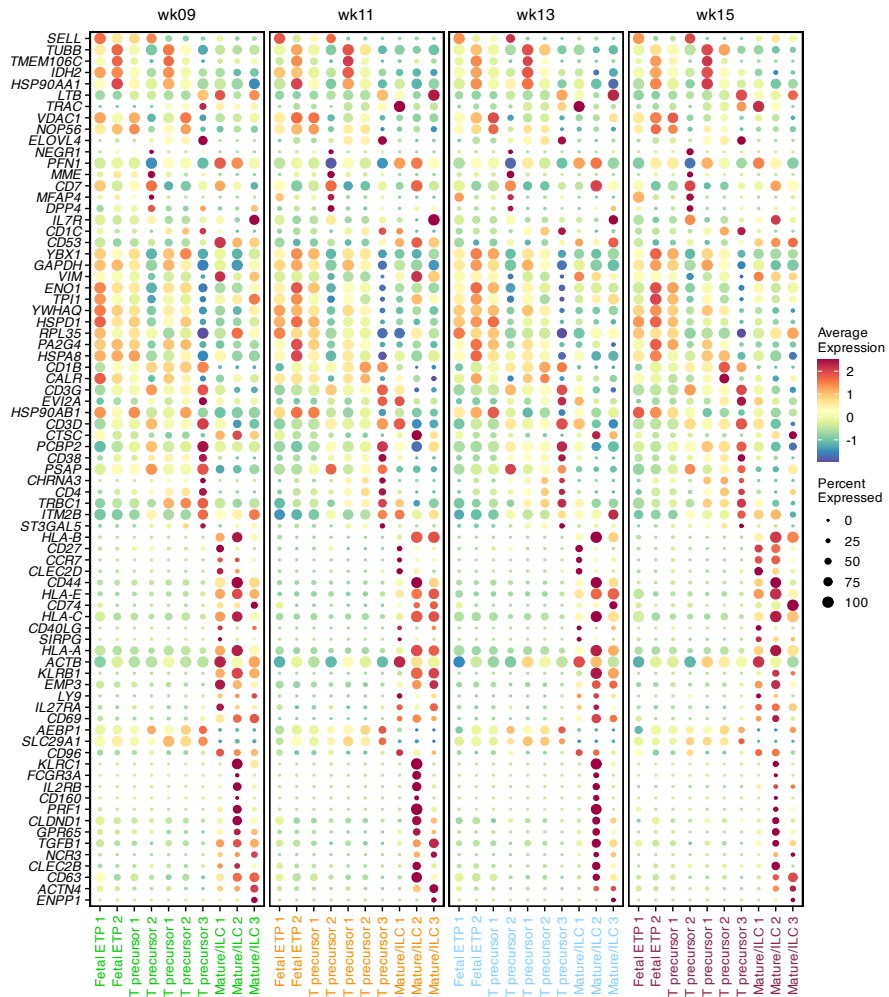

Supplement: Supplementary file 12 [file Data_Sheet_8.PDF]

**A**

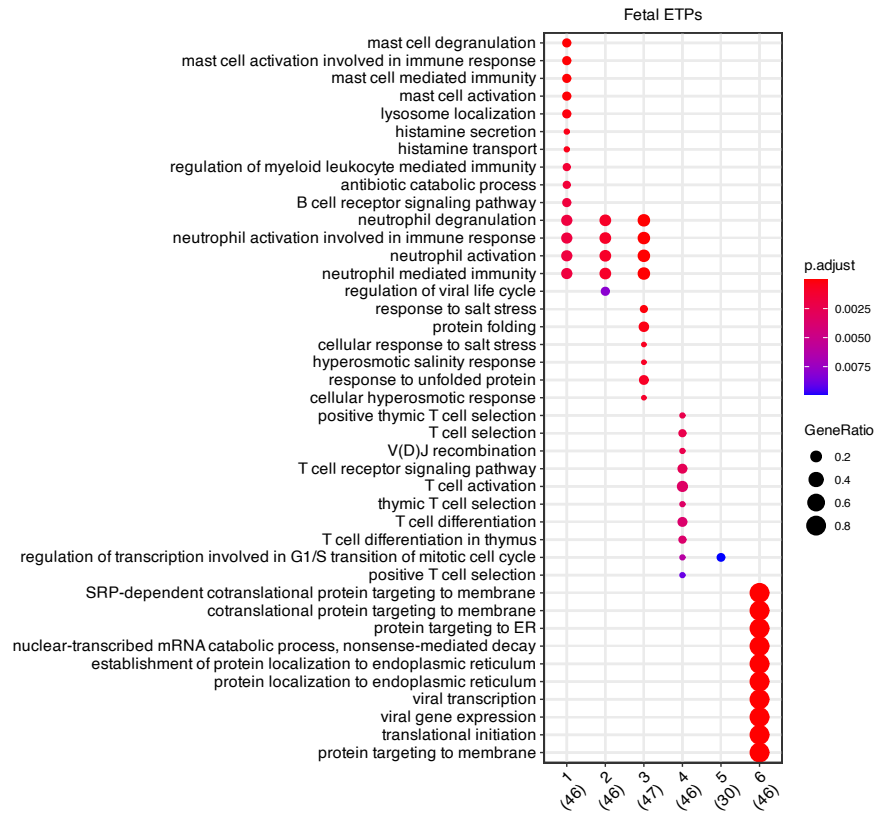

**B**

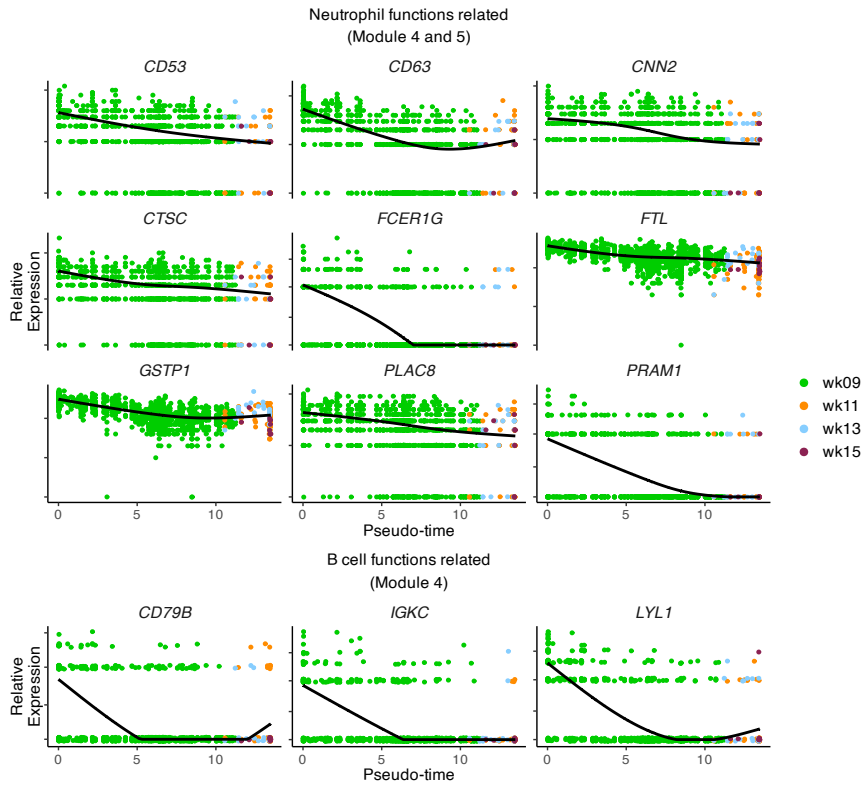

Supplement: Supplementary file 13 [file Data_Sheet_9.PDF]

**A**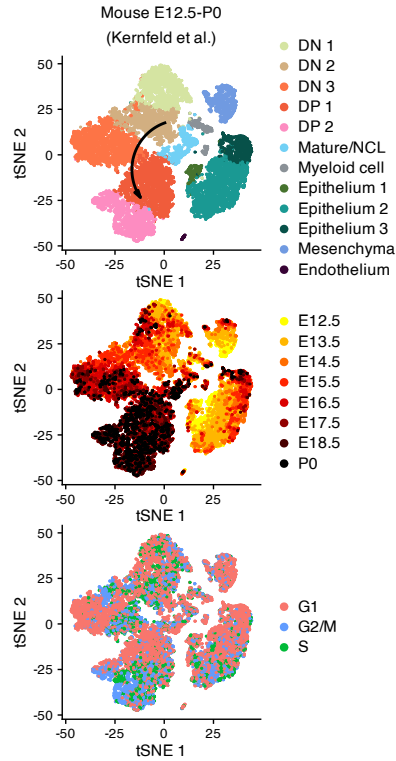**B**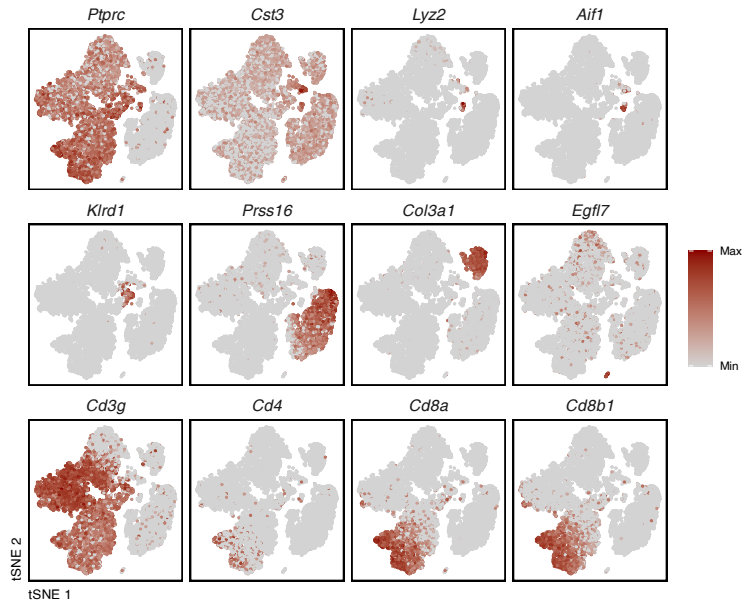**C**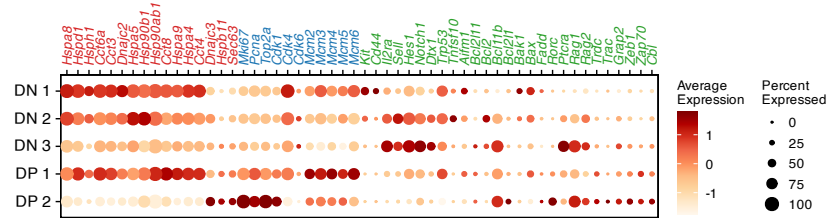**D**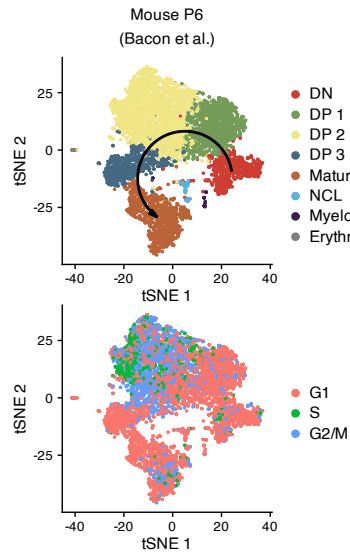**E**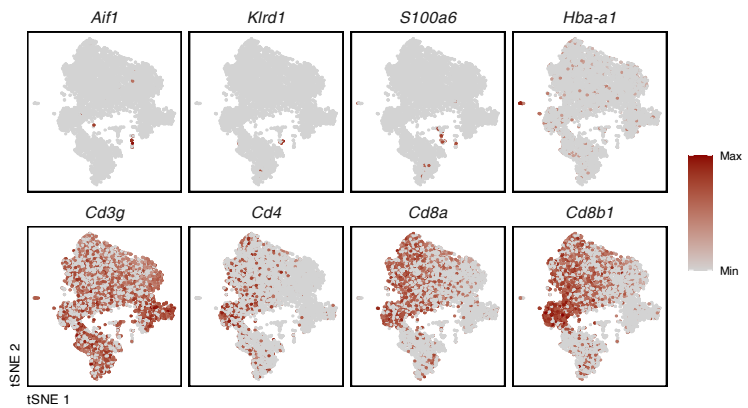**F**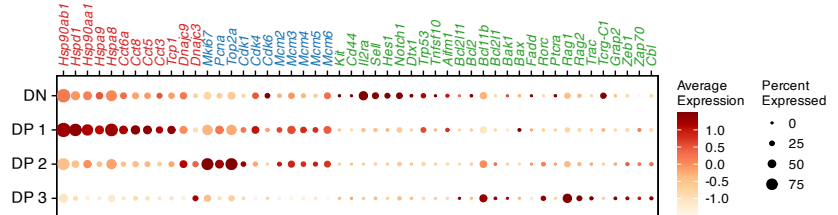

Supplement: Supplementary file 14 [file Data_Sheet_10.PDF]

**A**

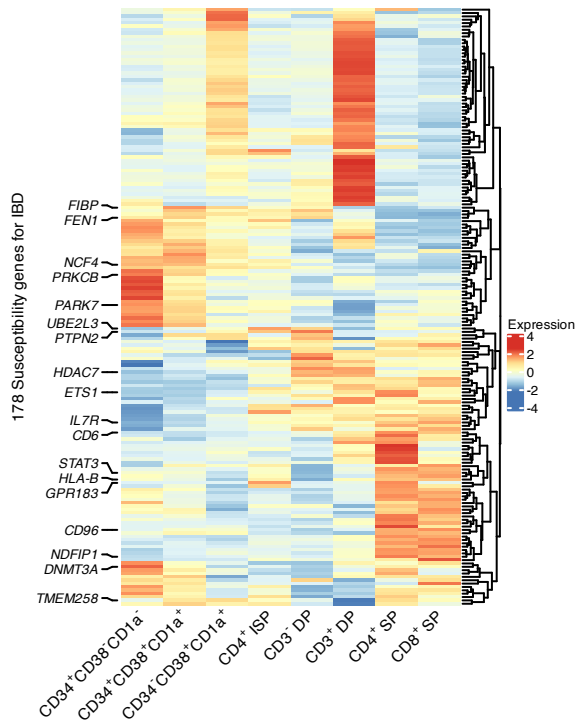

**B**

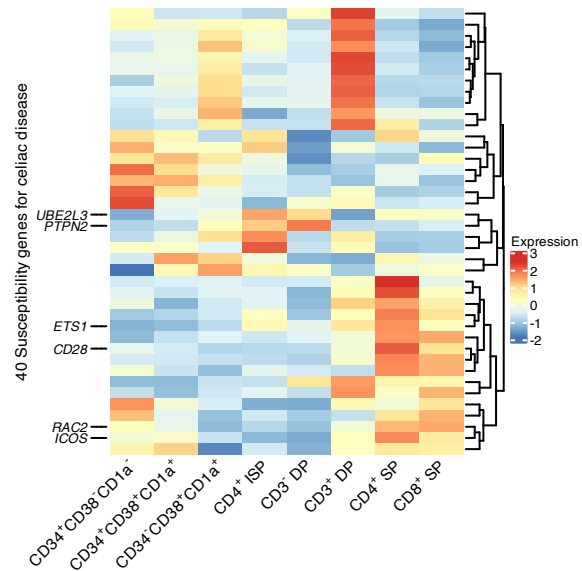

**C**

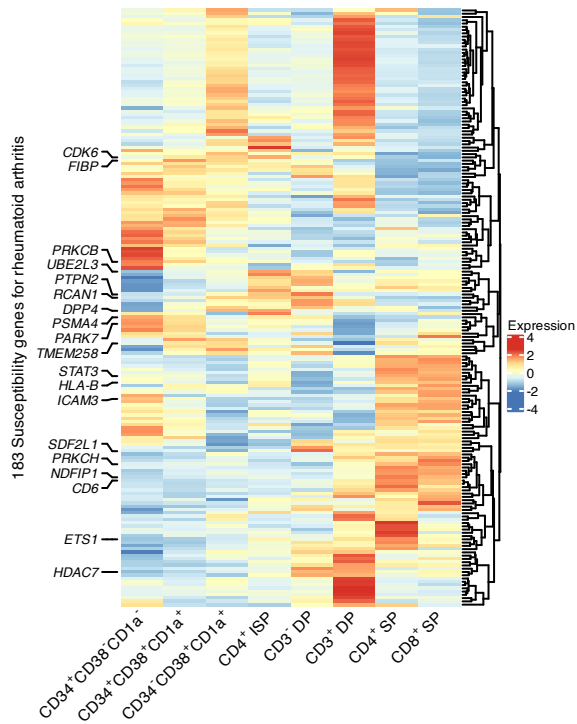

**D**

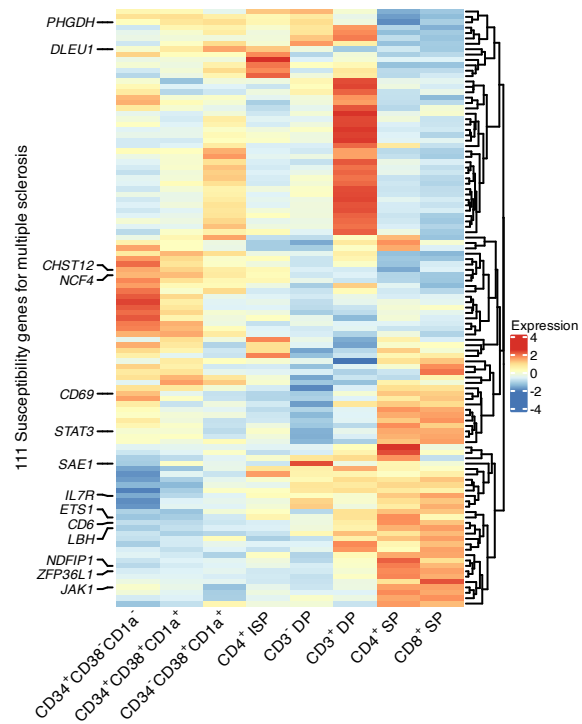

Supplement: Supplementary file 16 [file Data_Sheet_12.PDF]
