# Supplementary material for: Integrative Single-Cell Transcriptomic Analysis of Human Fetal Thymocyte Development
Source: Front Genet. 2021 Jul 2;12:679616. doi: 10.3389/fgene.2021.679616 (PMC8284395; doi:10.3389/fgene.2021.679616)
Supplement: Supplementary file 9 [file Data_Sheet_5.PDF]

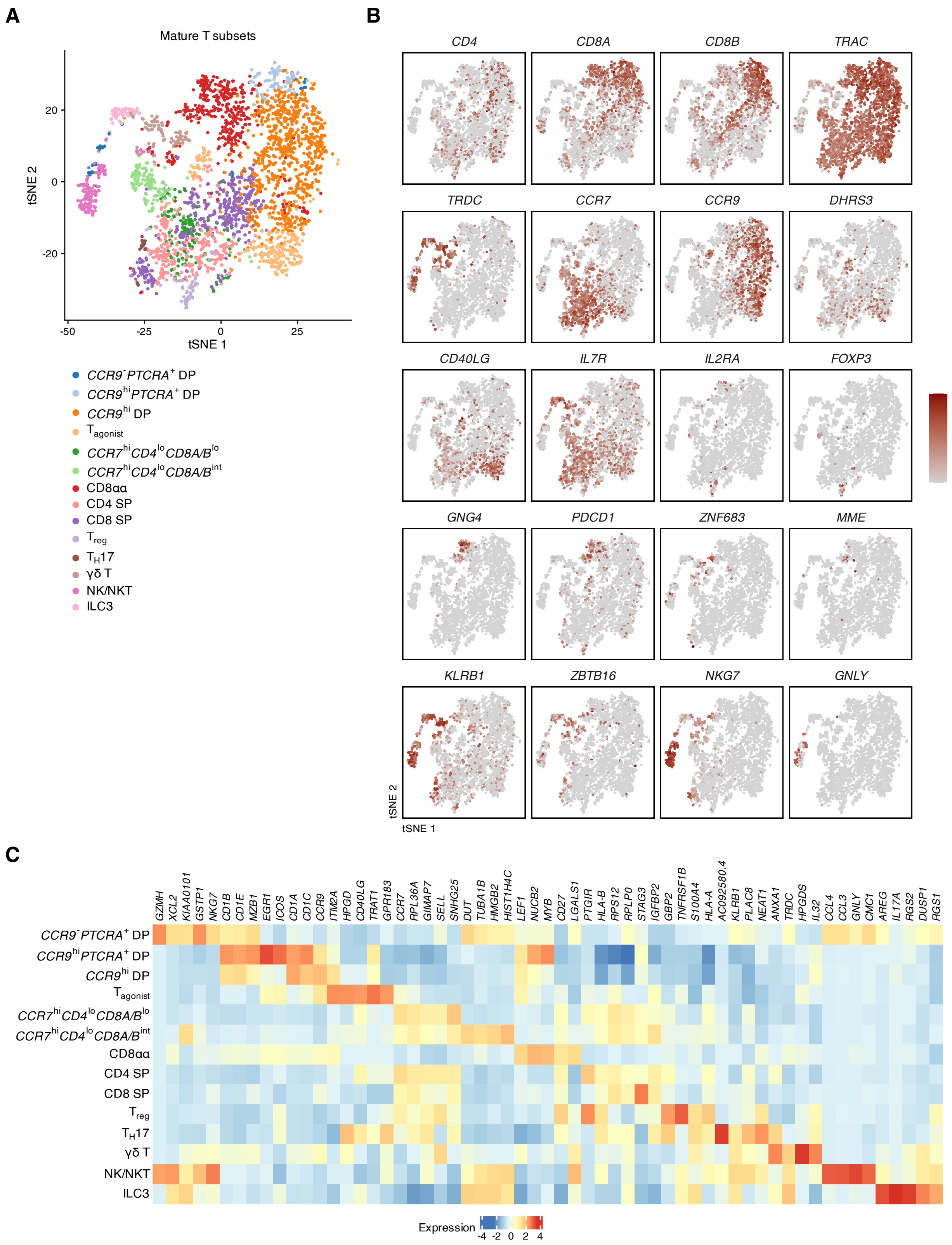
A
Mature T subsets
tSNE 2
tSNE 1
20
0
-20
-50
-25
0
25
CCR9⁻PTCRA⁺ DP
CCR9hiPTCRA⁺ DP
CCR9hi DP
Tagonist
CCR7hiCD4loCD8A/Blo
CCR7hiCD4loCD8A/Bint
CD8αα
CD4 SP
CD8 SP
Treg
TH17
γδ T
NK/NKT
ILC3
B
CD4
CD8A
CD8B
TRAC
TRDC
CCR7
CCR9
DHRS3
CD40LG
IL7R
IL2RA
FOXP3
Max
Min
GNG4
PDCD1
ZNF683
MME
KLRB1
ZBTB16
NKG7
GNLY
tSNE 2
tSNE 1
C
GZMH XCL2 KIAA0101 GSTP1 NKG7 CD1B CD1E MZB1 EGR1 ICOS CD1A CD1C CCR9 ITM2A HPGD CD40LG TRAT1 GPR183 CCR7 RPL36A GIMAP7 SELL SNHG25 DUT TUBA1B HMGB2 HIST1H4C LEF1 NUCB2 MYB CD27 LGALS1 PTGIR HLA-B RPS12 RPLP0 STAG3 IGFBP2 GBP2 TNFRSF1B S100A4 HLA-A AC092580.4 KLRB1 PLAC8 NEAT1 ANXA1 TRDC HPGDS IL32 CCL4 CCL3 GNLY CMC1 AREG IL17A RGS2 DUSP1 RGS1
CCR9⁻PTCRA⁺ DP
CCR9hiPTCRA⁺ DP
CCR9hi DP
Tagonist
CCR7hiCD4loCD8A/Blo
CCR7hiCD4loCD8A/Bint
CD8αα
CD4 SP
CD8 SP
Treg
TH17
γδ T
NK/NKT
ILC3
Expression
-4 -2 0 2 4
