# Supplementary material for: Integrative Single-Cell Transcriptomic Analysis of Human Fetal Thymocyte Development
Source: Front Genet. 2021 Jul 2;12:679616. doi: 10.3389/fgene.2021.679616 (PMC8284395; doi:10.3389/fgene.2021.679616)
Supplement: Supplementary file 15 [file Data_Sheet_11.PDF]

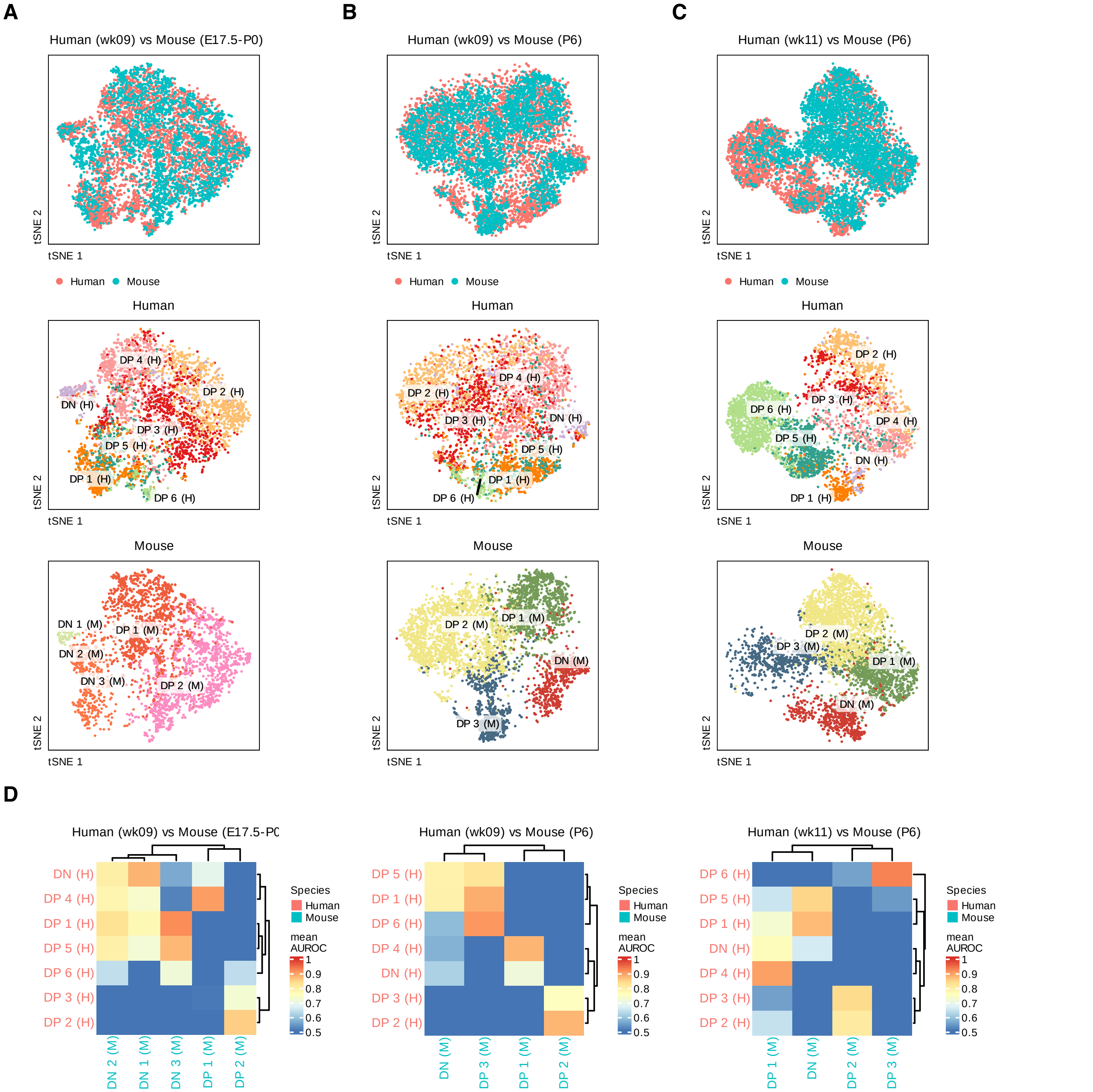

A
Human (wk09) vs Mouse (E17.5-P0)
B
Human (wk09) vs Mouse (P6)
C
Human (wk11) vs Mouse (P6)
tSNE 1
tSNE 2
Human
Mouse
DP 4 (H)
DP 2 (H)
DN (H)
DP 3 (H)
DP 5 (H)
DP 1 (H)
DP 6 (H)
DN 1 (M)
DP 1 (M)
DN 2 (M)
DN 3 (M)
DP 2 (M)
DP 3 (M)
DN (M)
D
Human (wk09) vs Mouse (E17.5-P0
Human (wk09) vs Mouse (P6)
Human (wk11) vs Mouse (P6)
Species
Human
Mouse
mean
AUROC
1
0.9
0.8
0.7
0.6
0.5
