# Supplementary material for: Integrative Single-Cell Transcriptomic Analysis of Human Fetal Thymocyte Development
Source: Front Genet. 2021 Jul 2;12:679616. doi: 10.3389/fgene.2021.679616 (PMC8284395; doi:10.3389/fgene.2021.679616)
Supplement: Supplementary file 17 [file Data_Sheet_13.docx]

**Supplementary Figure 1. Removal of ambient mRNAs and putative doublets.** (**A**) Initial clustering and expression of hemoglobin genes within each sample. (**B**) Gene annotations for ambient mRNAs removed by SoupX. (**C**) Visualization of the initial clustering (top) and putative doublets (bottom) in the raw data merging the four samples. (**D**) Heatmap of the expression of cell-type markers in doublets. (**E**) Violin plots showing metrics for quality-control within each dataset.

**Supplementary Figure 2. The heterogeneity of lymphocytes and non-lymphocytes identified in human the thymus.** (**A**) Visualization of the initial clustering (top) and fetal stages (bottom) within the cleaned data merging four samples. (**B**) Expression of cell-type marker genes in t-SNE plots corresponding to **A**. (**C**) Heatmap of DEGs among cell populations corresponding to **A**.

**Supplementary Figure 3. Initial clustering analysis of thymocytes from each sample.** (**A**) Visualization of the initial clustering of thymocytes from each sample with t-SNE. (**B**) Expression of marker genes related to thymocyte differentiation in t-SNE plots corresponding to **A**. (**C**) The number of detected genes and the expression of stress-related genes in t-SNE plots corresponding to **A**.

**Supplementary Figure 4. Exploration of differentiating thymocytes.** (**A**) Visualization of differentiating thymocytes corresponding to Figure 1A, divided by fetal stages. (**B**) Expression of marker genes visualized with t-SNE plots corresponding to Figure 1A. (**C**) Heatmap of DEGs among cell populations corresponding to **A**. (**D**) Heatmap of DEGs corresponding to **C** in sorted thymocytes.

**Supplementary Figure 5. Exploration of mature thymocytes.** (**A**) Visualization of mature thymocytes corresponding to Figure 1E. (**B**) Expression of marker genes visualized with t-SNE plots corresponding to Figure 1, E and F. (**C**) Heatmap of DEGs among cell populations corresponding to **A**.

**Supplementary Figure 6. Subpopulations of conventional SPs and comparison between single-cell and bulk data.** (**A**) Heatmap showing the expression of DEGs among cell populations corresponding to Figure 1G. (**B**) Heatmap of the same DEGs in **A** in microarray data of sorted SPs. (**C**) Significant enrichment of BP terms within SP subpopulations.

**Supplementary Figure 7. Analysis of published scRNA-seq data of the early fetal thymus.** (**A**) Initial clustering of hematopoietic and non-hematopoietic cells. (**B**) Expression of cell-type genes visualized with t-SNE plots corresponding to **A**. (**C**) Sub-clustering for hematopoietic cells. (**D**) Expression of specific genes visualized with t-SNE plots corresponding to **C**. (**E**) Expression of TCR and several marker genes in subsets of hematopoietic cells. (**F**) Dot plots of conserved DEGs identified in Figure 2C among subsets of hematopoietic cells.

**Supplementary Figure 8. Expression of stage-conserved DEGs encoding transcription factors (TFs) and surface proteins related to αβ and γδ precursors.** (**A**) Dot plots showing conserved DEGs encoding TFs. (**B**) Dot plots showing conserved DEGs encoding surface proteins.

**Supplementary Figure 9. Gene modules altered along with the developmental trajectory of ETPs.** (**A**) Significant BP terms altered during development of ETPs. (**B**) Expression of non-T lineage genes across pseudotime scores.

**Supplementary Figure 10. Clustering analysis of thymocytes from embryonic and postnatal mice.** (**A**) Visualization of the thymus of embryonic mice, colored by cell identities (top), embryonic days (middle) and cell-cycle phases (bottom). (**B**) Expression of cell-type markers in t-SNE plots corresponding to **A**. (**C**) Dot plots of homologous gene sets similar to Figure 1C in embryonic mouse. (**E**, **F** and **G**) Reanalysis of the thymus of postnatal mice, similar to **A**, **B** and **C** for embryonic mice.

**Supplementary Figure 11. Comparison of human fetuses (weeks 9 and 11) and mice regarding thymocyte differentiation.** (**A**) Integrating thymocytes of humans at week 9 with mice at E17.5-P0. (**B**) Integrating thymocytes of humans at week 9 with mice at P6. (**C**) Integrating thymocytes of humans at week 11 with mice at P6. (**D**) Heatmap of AUROC curve scores during integration analysis in **A** (left), **B** (middle), and **C** (right).

**Supplementary Figure 12. Expression of GWAS risk genes for human autoimmune disorders in sorted thymocytes.** (**A**) Heatmap of 178 susceptibility genes for IBD in sorted thymocytes. (**B**) Heatmap of 40 susceptibility genes for celiac disease in sorted thymocytes. (**C**) Heatmap of 183 susceptibility genes for rheumatoid arthritis in sorted thymocytes. (**D**) Heatmap of 111 susceptibility genes for MS in sorted thymocytes.

**Supplementary Table 1. Information of the scRNA-seq samples collected from fetal thymus of gestational week 9-15.**

**Supplementary Table 2. Conserved differentially-expressed genes of thymocyte identities transferred from reference dataset of human early thymus.**

**Supplementary Table 3. Differentially-expressed genes along with the pseudotime scores of fetal ETPs.**

**Supplementary Table 4. Conserved differentially-expressed genes characterized DNs and DPs in humans and mice.**
