# Supplementary material for: Integrative Single-Cell Transcriptomic Analysis of Human Fetal Thymocyte Development
Source: Front Genet. 2021 Jul 2;12:679616. doi: 10.3389/fgene.2021.679616 (PMC8284395; doi:10.3389/fgene.2021.679616)
Supplement: Supplementary file 6 [file Data_Sheet_2.PDF]

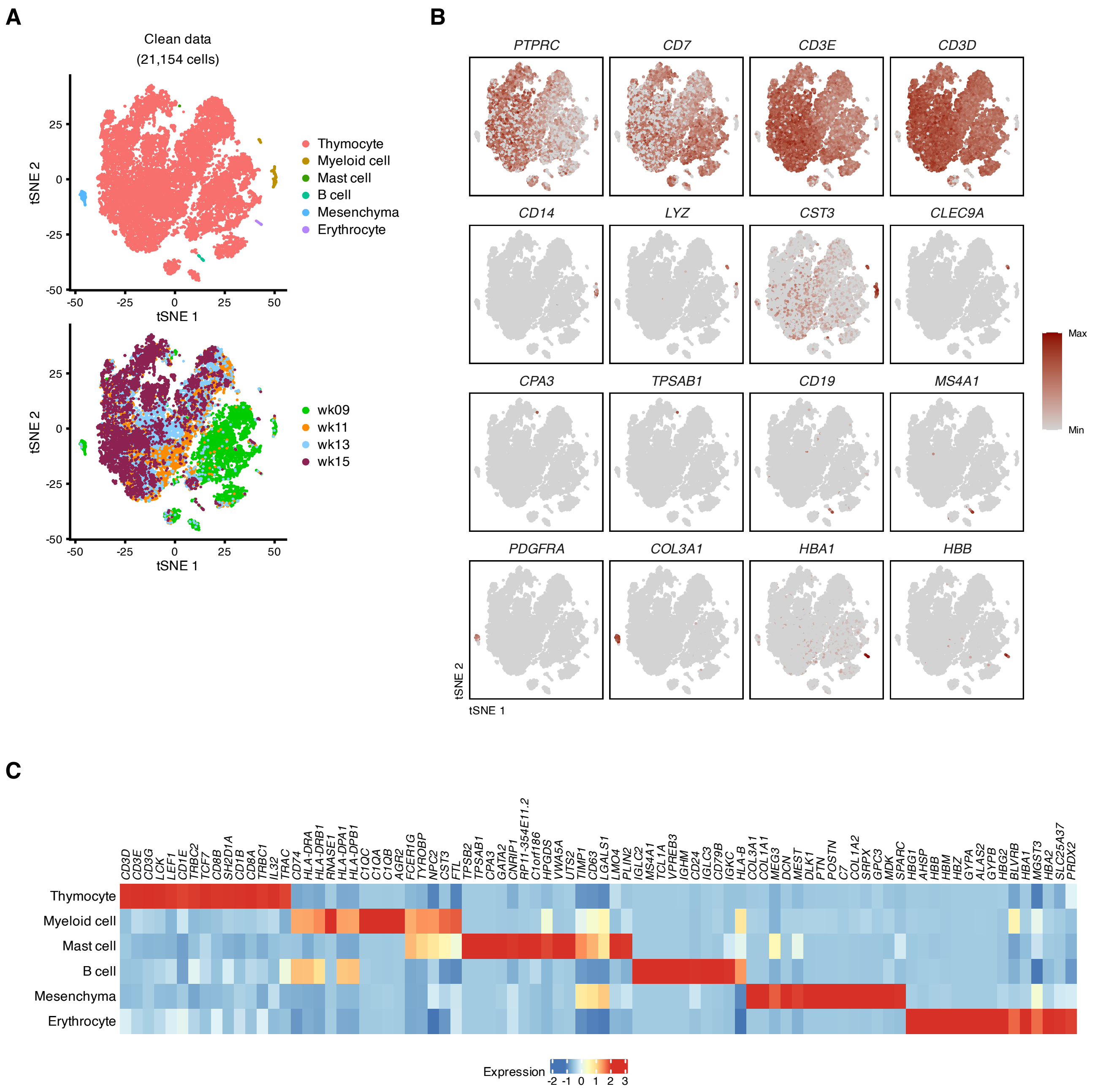

A
Clean data
(21,154 cells)
tSNE 2
tSNE 1
25
0
-25
-50
50
Thymocyte
Myeloid cell
Mast cell
B cell
Mesenchyma
Erythrocyte
wk09
wk11
wk13
wk15
B
PTPRC
CD7
CD3E
CD3D
CD14
LYZ
CST3
CLEC9A
CPA3
TPSAB1
CD19
MS4A1
PDGFRA
COL3A1
HBA1
HBB
Max
Min
C
CD3D CD3E CD3G LCK LEF1 CD1E TRBC2 TCF7 CD8B SH2D1A CD1B CD8A TRBC1 IL32 TRAC CD74 HLA-DRA HLA-DRB1 RNASE1 HLA-DPA1 HLA-DPB1 C1QC C1QA C1QB AGR2 FCER1G TYROBP NPC2 CST3 FTL TPSB2 TPSAB1 CPA3 GATA2 CNRIP1 RP11-354E11.2 C1orf186 HPGDS VWA5A UTS2 TIMP1 CD63 LGALS1 LMO4 PLIN2 IGLC2 MS4A1 TCL1A VPREB3 IGHM CD24 IGLC3 CD79B IGKC HLA-B COL3A1 COL1A1 MEG3 DCN MEST DLK1 PTN POSTN C7 COL1A2 SRPX GPC3 MDK SPARC HBG1 AHSP HBB HBM HBZ GYPA ALAS2 GYPB HBG2 BLVRB HBA1 MGST3 HBA2 SLC25A37 PRDX2
Expression
-2 -1 0 1 2 3
