# Supplementary material for: Integrative Single-Cell Transcriptomic Analysis of Human Fetal Thymocyte Development
Source: Front Genet. 2021 Jul 2;12:679616. doi: 10.3389/fgene.2021.679616 (PMC8284395; doi:10.3389/fgene.2021.679616)
Supplement: Supplementary file 8 [file Data_Sheet_4.PDF]

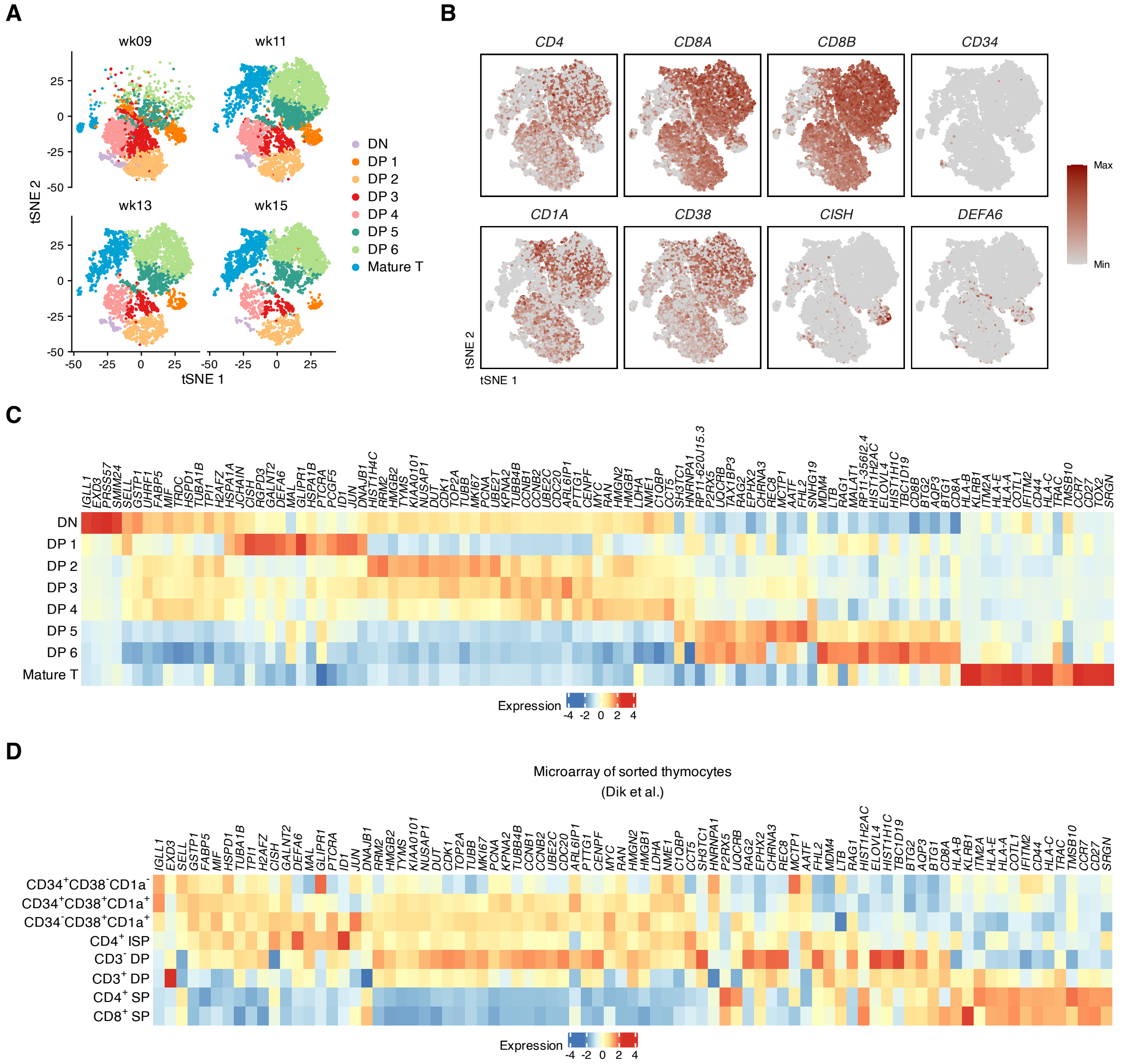
A
wk09
wk11
wk13
wk15
tSNE 2
tSNE 1
25
0
-25
-50
-50 -25 0 25
DN
DP 1
DP 2
DP 3
DP 4
DP 5
DP 6
Mature T
B
CD4
CD8A
CD8B
CD34
CD1A
CD38
CISH
DEFA6
Max
Min
tSNE 2
tSNE 1
C
IGLL1 EXD3 PRSS57 SMIM24 SELL GSTP1 UHRF1 FABP5 MIF TRDC HSPD1 TUBA1B TPI1 H2AFZ HSPA1A JCHAIN CISH RGPD3 GALNT2 DEFA6 MAL GLIPR1 HSPA1B PTCRA PCGF5 ID1 JUN DNAJB1 HIST1H4C RRM2 HMGB2 TYMS KIAA0101 NUSAP1 DUT CDK1 TOP2A TUBB MKI67 PCNA UBE2T KPNA2 TUBB4B CCNB1 CCNB2 UBE2C CDC20 ARL6IP1 PTTG1 CENPF MYC RAN HMGN2 HMGB1 LDHA NME1 C1QBP CCT5 SH3TC1 HNRNPA1 RP11-620J15.3 P2RX5 UQCRB TAX1BP3 RAG2 EPHX2 CHRNA3 REC8 MCTP1 AATF FHL2 SNHG19 MDM4 LTB RAG1 MALAT1 RP11-356I2.4 HIST1H2AC ELOVL4 HIST1H1C TBC1D19 CD8B BTG2 AQP3 BTG1 CD8A HLA-B KLRB1 ITM2A HLA-E HLA-A IFITM2 CD44 HLA-C TRAC TMSB10 CCR7 CD27 TOX2 SRGN
DN
DP 1
DP 2
DP 3
DP 4
DP 5
DP 6
Mature T
Expression
-4 -2 0 2 4
D
Microarray of sorted thymocytes
(Dik et al.)
IGLL1 EXD3 SELL GSTP1 FABP5 MIF HSPD1 TUBA1B TPI1 H2AFZ CISH GALNT2 DEFA6 MAL GLIPR1 PTCRA ID1 JUN DNAJB1 RRM2 HMGB2 TYMS KIAA0101 NUSAP1 DUT CDK1 TOP2A TUBB MKI67 PCNA KPNA2 TUBB4B CCNB1 CCNB2 UBE2C CDC20 ARL6IP1 PTTG1 CENPF MYC RAN HMGN2 HMGB1 LDHA NME1 C1QBP CCT5 SH3TC1 HNRNPA1 P2RX5 UQCRB RAG2 EPHX2 CHRNA3 REC8 MCTP1 AATF FHL2 MDM4 LTB RAG1 HIST1H2AC ELOVL4 HIST1H1C TBC1D19 BTG2 AQP3 BTG1 CD8A HLA-B KLRB1 ITM2A HLA-E HLA-A COTL1 IFITM2 CD44 HLA-C TRAC TMSB10 CCR7 CD27 SRGN
CD34+CD38-CD1a-
CD34+CD38+CD1a+
CD34-CD38+CD1a+
CD4+ ISP
CD3- DP
CD3+ DP
CD4+ SP
CD8+ SP
Expression
-4 -2 0 2 4
