# Supplementary material for: Integrative Single-Cell Transcriptomic Analysis of Human Fetal Thymocyte Development
Source: Front Genet. 2021 Jul 2;12:679616. doi: 10.3389/fgene.2021.679616 (PMC8284395; doi:10.3389/fgene.2021.679616)
Supplement: Supplementary file 11 [file Data_Sheet_7.PDF]

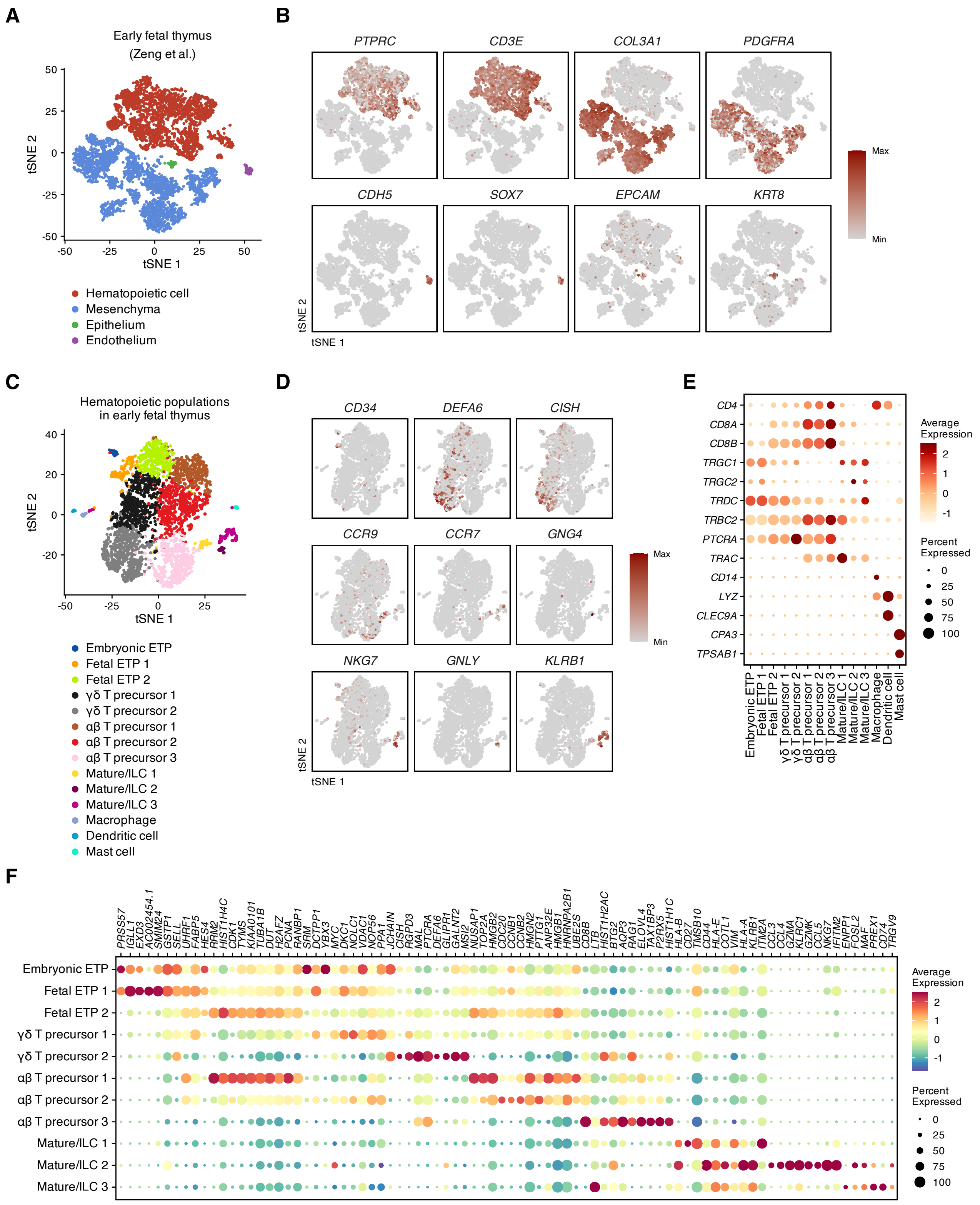

A
Early fetal thymus
(Zeng et al.)
tSNE 2
tSNE 1
50
25
0
-25
-50
-50
-25
0
25
50
Hematopoietic cell
Mesenchyma
Epithelium
Endothelium
B
PTPRC
CD3E
COL3A1
PDGFRA
CDH5
SOX7
EPCAM
KRT8
Max
Min
tSNE 2
tSNE 1
C
Hematopoietic populations
in early fetal thymus
tSNE 2
tSNE 1
40
20
0
-20
-50
-25
0
25
Embryonic ETP
Fetal ETP 1
Fetal ETP 2
γδ T precursor 1
γδ T precursor 2
αβ T precursor 1
αβ T precursor 2
αβ T precursor 3
Mature/ILC 1
Mature/ILC 2
Mature/ILC 3
Macrophage
Dendritic cell
Mast cell
D
CD34
DEFA6
CISH
CCR9
CCR7
GNG4
NKG7
GNLY
KLRB1
Max
Min
tSNE 2
tSNE 1
E
CD4
CD8A
CD8B
TRGC1
TRGC2
TRDC
TRBC2
PTCRA
TRAC
CD14
LYZ
CLEC9A
CPA3
TPSAB1
Embryonic ETP
Fetal ETP 1
Fetal ETP 2
γδ T precursor 1
γδ T precursor 2
αβ T precursor 1
αβ T precursor 2
αβ T precursor 3
Mature/ILC 1
Mature/ILC 2
Mature/ILC 3
Macrophage
Dendritic cell
Mast cell
Average Expression
2
1
0
-1
Percent Expressed
0
25
50
75
100
F
PRSS57 IGLL1 EXD3 AC002454.1 SMIM24 GSTP1 SELL UHRF1 FABP5 HES4 RRM2 HIST1H4C CDK1 TYMS KIAA0101 TUBA1B DUT H2AFZ PCNA RANBP1 SRM DCTPP1 YBX3 MYC DKC1 NOLC1 VDAC1 NOP56 PPA1 JCHAIN CISH RGPD3 MAL PTCRA DEFA6 GLIPR1 GALNT2 MSI2 NUSAP1 TOP2A HMGB2 CDC20 CCNB1 CCNB2 HMGN2 PTTG1 ANP32E HMGB1 HNRNPA2B1 UBE2S CD8B LTB HIST1H2AC BTG2 AQP3 RAG1 ELOVL4 TAX1BP3 P2RX5 HIST1H1C HLA-B CD27 TMSB10 CD44 HLA-E COTL1 VIM HLA-A KLRB1 ITM2A CCL3 CCL4 GZMA KLRC1 GZMK CCL5 NKG7 IFITM2 ENPP1 FOSL2 MAF PREX1 CD74 TRGV9
Embryonic ETP
Fetal ETP 1
Fetal ETP 2
γδ T precursor 1
γδ T precursor 2
αβ T precursor 1
αβ T precursor 2
αβ T precursor 3
Mature/ILC 1
Mature/ILC 2
Mature/ILC 3
Average Expression
2
1
0
-1
Percent Expressed
0
25
50
75
100
